# Cortical dynamics of icon perception: effects of concreteness and attractiveness

**DOI:** 10.1093/cercor/bhag075

**Published:** 2026-06-22

**Authors:** Jiaqi Zheng, Weiyong Xu, Johanna Silvennoinen, Fengyu Cong, Tiina Parviainen, Tuomo Kujala

**Affiliations:** Faculty of Information Technology, University of Jyväskylä, Mattilanniemi 2, P.O. Box 35, FI-40014, Jyväskylä, Finland; School of Biomedical Engineering, Faculty of Medicine, Dalian University of Technology, Dalian 116024, Liaoning Province, China; Centre for Interdisciplinary Brain Research, University of Jyväskylä, Mattilanniemi 6, FI-40014, Jyväskylä, Finland; Centre for Interdisciplinary Brain Research, University of Jyväskylä, Mattilanniemi 6, FI-40014, Jyväskylä, Finland; Department of Psychology, University of Jyväskylä, PO BOX 35, Mattilanniemi 6, FI-40014, Jyväskylä, Finland; Faculty of Information Technology, University of Jyväskylä, Mattilanniemi 2, P.O. Box 35, FI-40014, Jyväskylä, Finland; Faculty of Information Technology, University of Jyväskylä, Mattilanniemi 2, P.O. Box 35, FI-40014, Jyväskylä, Finland; School of Biomedical Engineering, Faculty of Medicine, Dalian University of Technology, Dalian 116024, Liaoning Province, China; Key Laboratory of Social Computing and Cognitive Intelligence, Dalian University of Technology, Ministry of Education, Dalian 116024, Liaoning Province, China; School of Software Engineering, Dalian University, Dalian 116622, Liaoning Province, China; Centre for Interdisciplinary Brain Research, University of Jyväskylä, Mattilanniemi 6, FI-40014, Jyväskylä, Finland; Department of Psychology, University of Jyväskylä, PO BOX 35, Mattilanniemi 6, FI-40014, Jyväskylä, Finland; Faculty of Information Technology, University of Jyväskylä, Mattilanniemi 2, P.O. Box 35, FI-40014, Jyväskylä, Finland

**Keywords:** attractiveness, concreteness, icon perception, magnetoencephalography (MEG), representational similarity analysis (RSA)

## Abstract

Icons, as simplified visual symbols, play a key role in visual communication, yet little neuroimaging research has addressed how icons are represented in the brain. We investigated how concreteness and attractiveness modulate the spatiotemporal dynamics of icon processing in a 2 × 2 factorial design in 35 adults using magnetoencephalography. Source-level event-related field (ERF) analysis and representational similarity analysis (RSA) were used to characterize neural responses, with partial RSA isolating each feature’s unique contribution after controlling for low-level visual similarity. ERF results showed that concreteness exerted a robust and sustained influence on neural dynamics, with concrete icons eliciting stronger responses than abstract ones from 90 to 1,000 ms, emerging in bilateral occipital cortices and extending to occipitotemporal, temporal, and parietal regions. RSA confirmed concreteness as a representational dimension across processing stages. Attractiveness showed an early but transient effect in occipital and ventral occipitotemporal regions (80 to 130 ms), though this did not survive RSA after controlling for low-level visual property models. A concreteness × attractiveness interaction modulated early occipital and later parietal processing (100 to 185 ms), indicating these features do not operate independently. Our results reveal that semantic content outweighs esthetic appeal in shaping the neural representation of icons.

## Introduction

In the digital age, icons have become the primary medium through which people interact with technology, serving as visual symbols that link visual recognition with semantic understanding (Dove 2009; Huang et al. 2015). By conveying complex meanings through minimal visual cues (Marcus 2003; Marr 2010; Collaud et al. 2022), icons enable efficient navigation of digital environments, leveraging the visual system’s ability to process perceptual information rapidly. Icon perception can be viewed as a unique intersection of visual object recognition, semantic interpretation, and esthetic evaluation, yet the neural mechanisms underlying how we perceive, evaluate, and respond to iconic stimuli remain poorly understood.

Visual object recognition is a core function of the human visual system, relying on rapid hierarchical processing along the ventral visual stream that transforms low-level visual features into stable object representations within a few 100 ms (Grill-Spector and Kanwisher 2005; DiCarlo et al. 2012). Neuroimaging research has shown that neural responses to iconic images differ markedly from those elicited by corresponding real-world objects, indicating sensitivity to visual abstraction and representational format (Shin et al. 2008). Early stages of visual processing involve the extraction of low-level structural properties such as edges, contrast, and coarse shape information, and are commonly linked to activity in early visual cortices such as V1 and V2 (Di Russo et al. 2002; Kuefner et al. 2010). As processing unfolds, more complex contours and shape components are integrated, and visual image processing extends beyond occipital cortex to a broader neural network including posterior temporal, parietal, and frontal regions that may support higher-order interpretation and evaluation (Grill-Spector et al. 2001; Rossion et al. 2003). Consistent with this view, functional magnetic resonance imaging (fMRI) studies of icon perception have reported recruitment not only in visual regions but also in frontal and parietal cortices, suggesting that icon processing extends beyond basic shape analysis (Huang et al. 2015). More direct temporal evidence from event-related potential (ERP) studies further suggests that icon processing unfolds across multiple stages. Early components appear to be sensitive to icon characteristics related to meaning accessibility. For example, icons whose meanings are more directly conveyed elicit larger N100 amplitudes, suggesting that meaning-related differences can influence early stages of processing (Cherng et al. 2016; Haider, 1964; Vogel and Luck, 2000). Other studies further indicate that icon understanding involves later semantic stages associated with semantic relevance and pictogram-semantic mapping structure. In particular, semantic incongruence has been linked to N400-related responses, and variations in pictogram-semantic mapping structure have also been shown to influence icon understanding and semantic processing fluency (Dong et al. 2023; Hou and Pan 2025). In addition, affective properties such as attractiveness have been linked to later attentional and evaluative responses, with more attractive icons eliciting larger P2, P3, and late positive potential amplitudes than less attractive icons (Anderson, 2013; Cao et al. 2021). Together, these findings suggest that icon processing is not a unitary process but involves multiple stages that may be shaped by the perceptual features of the icons themselves, among which concreteness and perceived attractiveness have emerged as 2 particularly noteworthy dimensions in influencing icon perception (Schröder and Ziefle 2008; McDougall and Isherwood 2009; Silvennoinen and Jokinen 2016).

Icon perceptual features are not limited to low-level visual properties (eg shape, size, and contour), but also reflect how humans mentally process and interpret icons, capturing cognitive attributes that determine how easily and effectively an icon can be perceived, understood, and remembered (Tung and Chen 2016; Jylhä and Hamari 2019). Concreteness has been considered a key factor in the effective understanding and recognition of icons (West and Holcomb 2000; Collaud et al. 2022; Zheng et al. 2026), and is defined as the extent to which an icon visually resembles a real-world object in representing a specific concept (McDougall et al. 1999). It is important to note that concreteness in the context of visual icons is conceptually distinct from lexical concreteness or imageability as traditionally defined in the cognitive and psycholinguistic literature. In those domains, concreteness refers to the extent to which a verbal stimulus denotes a tangible entity or evokes a mental image (Paivio et al. 1968; Brysbaert et al. 2014), and its effects are typically associated with late-stage semantic processing and conceptual retrieval (Binder et al. 2005; Vigliocco et al. 2009; Barber et al. 2013). In contrast, icon concreteness in the present study is operationalized as a perceptual resemblance dimension, reflecting the degree to which an icon’s visuospatial structure resembles a real-world object or entity. As a visual-perceptual construct, icon concreteness is not limited to purely semantic or linguistic representations, but is grounded in the direct mapping between an icon’s visual form and stored mental representations of real-world objects (McDougall and Isherwood 2009). Representations with higher concreteness tend to provide clearer visual cues that afford more direct perceptual decoding, thereby supporting faster and more accurate recognition (McDougall et al. 1999; Isherwood et al. 2007). An ERP study further suggests that concrete icons are processed more efficiently during early stages of recognition, as reflected by smaller neural responses and shorter response times (Xiao-Fan et al. 2014). This operationalization is consistent with accounts of visual object recognition that emphasize the role of structural descriptions in matching visual input to stored representations (Watt et al. 1983; Biederman 1987). Because perceptual resemblance provides a more direct mapping between visual form and stored object representations, it may facilitate not only early perceptual encoding but also subsequent access to associated semantic knowledge (Cherng et al. 2016). Icon concreteness may therefore engage neural processing across multiple stages, with perceptual resemblance to real-world objects supporting early visual encoding and semantic interpretation emerging at later stages.

However, icons as visual elements in user interfaces are not purely functional, as their perceived esthetic appeal can also shape users’ perceptions and influence interaction (Jylhä and Hamari 2019). Perceived attractiveness refers to the extent to which an icon is judged as visually pleasing or appealing by viewers and represents another important dimension of icon perception (Tractinsky et al. 2000), typically assessed through subjective liking or esthetic pleasantness ratings that remain relatively stable within individuals over time (Bargas-Avila and Hornbæk 2011; Silvennoinen and Jokinen 2016; Jacobsen and Beudt 2017). Attractive icons have been shown to elicit stronger attentional engagement, greater user preference, and heightened affective responses, and tend to be evaluated more positively and capture attention more effectively (Tuch et al. 2012; Reppa and McDougall 2015; Sutcliffe 2022). These behavioral findings suggest that the influence of attractiveness is unlikely to be limited to explicit preference judgments alone, but may emerge across multiple stages of visual processing. Neuroesthetics accounts similarly propose that esthetic experience does not arise solely at a late evaluative stage, but is shaped by the interaction of sensory processing, affective valuation, and meaning-related systems (Chatterjee and Vartanian 2014; Pearce et al. 2016). Although direct cognitive neuroscience evidence on icon attractiveness remains limited, icons that possess esthetic appeal may likewise engage not only later evaluative responses, but also earlier processes related to attentional orienting and perceptual selection. In this sense, attractiveness may function not merely as an outcome of icon perception but as a factor that helps shape how icons are processed from early perception to later esthetic appraisal.

In addition, icon perception may also be modulated by familiarity, defined as the frequency with which a user has previously encountered an icon (Krueger 1975; McDougall and Isherwood 2009; Hu et al. 2023). Familiarity can facilitate recognition and memory retrieval, leading to more efficient processing of visual symbols. In the context of icons, greater familiarity may also support the mapping between visual symbols and their associated meanings, thereby facilitating conceptual interpretation (McDougall et al. 1999). Prior studies have shown that familiarity with interface elements influences both icon recognition performance and esthetic evaluation (McDougall et al. 2016; Shen et al. 2020). Given that familiarity naturally varies across icons and individuals and may shape perceptual and evaluative processing, we included familiarity as a measured experiential factor and treated it as an exploratory dimension to examine whether and how prior exposure shapes the neural representational structure of icons. In summary, concreteness may facilitate perceptual decoding through clearer visual cues and stronger resemblance to real-world objects, whereas attractiveness may enhance attention capture and early perceptual processing, and familiarity, as an experiential factor, may support recognition and facilitate the mapping between visual symbols and their associated meanings. Taken together, these findings suggest that concreteness and attractiveness may influence neural processing through partially distinct pathways, and that their effects may not be strictly independent.

Despite extensive behavioral research on icon perception, such approaches can primarily reveal processing outcomes, rather than how icon features are dynamically represented in the brain. ERP studies have provided important insights into the temporal stages of icon perception, but they mainly characterize differences in response amplitude and latency at the sensor level, offering limited information about the spatial organization and representational structure of neural processes. It therefore remains unclear how perceptual features such as concreteness and attractiveness are encoded within distributed neural activity patterns, and how these representations unfold across cortical regions over time. This question is particularly relevant because icon perception likely recruits distributed cortical networks that extend beyond early visual areas to temporal, parietal, and frontal regions. Magnetoencephalography (MEG) provides millisecond-level temporal resolution together with improved spatial characterization of cortical sources (Hämäläinen et al. 1993; Baillet 2017), making it well suited to track the spatiotemporal dynamics of icon perception. In the present study, we used MEG to examine the spatiotemporal progression of neural activity evoked by icons that systematically varied in concreteness and attractiveness. We analyzed event-related fields (ERFs) to characterize the temporal dynamics of icon processing and conducted source localization to estimate the approximate cortical locations of the observed effects.

To further examine how multiple icon features are represented in neural activity patterns during stimulus processing, we employed representational similarity analysis (RSA; Kriegeskorte et al. 2008) to investigate the neural representation of key perceptual features of icons, which enables the examination of whether and when specific icon features organize neural representational geometry across time and cortex. RSA allows for the comparison of representational geometries between different data modalities by constructing representational dissimilarity matrices (RDMs) derived from theoretical models, behavioral measurements, and neural responses. Examining neural similarity structures at the single-trial level enables us to determine whether perceptual dimensions of icons are encoded in neural activity patterns across time and whether the representational structure of subjective evaluations is reflected in these neural responses (Cichy et al. 2014; Hebart et al. 2018; Xu et al. 2024). By correlating behavioral and neural RDMs, we aim to identify the time periods and brain regions in which neural representations correspond to behaviorally relevant representational structure. We hypothesized that concreteness, as a key determinant of how readily perceptual features can be interpreted, would modulate neural responses across early perceptual encoding, mid-level structural integration, and later interpretive stages. Attractiveness was expected to modulate early stages of visual encoding and to be involved in later stages of esthetic evaluation. We additionally explored whether concreteness and attractiveness interact during icon perception, given that both features may jointly influence early perceptual encoding, without assuming a specific direction or functional role for this interaction. Within the RSA framework, we anticipated that concreteness would exhibit a sustained representational correspondence spanning early to late processing stages, consistent with its role across multiple levels of visual and semantic processing. Attractiveness was expected to show a representational signature primarily at early stages, though whether it is also involved in later evaluative stages remains an open question. Familiarity was included as an exploratory dimension; based on its grounding in memory and prior experience, familiarity-related representational effects, if present, were expected to emerge primarily at later processing stages associated with memory retrieval and recognition, rather than during early perceptual encoding.

## Materials and methods

### Participants

To obtain a well-characterized set of experimental stimuli, we first recruited 75 participants to complete a concreteness rating survey (7-point Likert scale). Because the full stimulus set was large and consisted of 6 icon categories, the survey was divided into 2 parts, with 3 categories rated in each part. Thirty-nine participants (18 males, 18 females, 1 nonbinary, and 2 preferred not to disclose [PND]; M = 29.8 yr, SD = 9.5) completed the first part and 36 participants (14 males, 17 females, 2 nonbinary, and 3 PND; M = 27.3 yr, SD = 8.6) completed the second part. Participation was compensated through a €10 gift card lottery. This sample was used solely for concreteness evaluation.

Second, we recruited a new group of participants (*n* = 64, 29 males, 35 females; M = 27.6 yr, SD = 6.6) to complete an attractiveness rating survey (7-point Likert scale). Because this sample also served as the eligibility pool for the subsequent MEG experiment, it was kept independent of the concreteness survey to avoid repeated exposure to the stimuli. During recruitment, participants were informed that they might later be invited to a separate MEG session and were required not to have taken part in the concreteness survey. Compensation was provided through a €10 gift card lottery. A demographic summary of the rating surveys is provided in Supplementary Table S1.

In total, 35 healthy adults (20 males, 14 females, 1 PND; M = 27.96 yr, SD = 6.91) took part in the MEG experiment. To minimize potential effects of stimulus familiarity, none of the MEG participants had previously taken part in the concreteness rating phase. All participants were screened for the following exclusion criteria: cardiovascular disease, significant sensory impairments (eg vision or hearing loss), neurological disorders, neurodevelopmental conditions (eg attention-deficit hyperactivity disorder [ADHD], dyslexia), neurodegenerative diseases (eg dementia), use of medications affecting the central nervous system, history of brain injury, and the presence of metal objects in the body, including dental braces and piercings. All participants had normal or corrected-to-normal vision, including 5 individuals who wore eyeglasses to achieve corrected vision. After completing the MEG session, participants filled out a familiarity rating survey. Each participant received a €20 gift card as compensation.

The study was conducted in accordance with the principles of the Declaration of Helsinki and was approved by the Ethics Committee of the University of Jyväskylä, Finland (1631/13.00.04.00/2022). All participants provided written informed consent prior to participation. Participation was voluntary, data were collected anonymously, and no identifying information was recorded.

### Stimuli

The concreteness rating survey was constructed based on 6 icon categories that the researchers selected, commonly used in contemporary digital interfaces: bookmarks, home, settings, profile, info, and search. A total of 420 icons were randomly collected from online repositories and converted to grayscale to ensure a uniform visual appearance. Participants were provided with clear written instructions, definitions of key terms, and an example image illustrating different levels of concreteness, which served as a visual anchor for the task. They were then asked to rate the degree of concreteness of each icon on a 7-point scale (1 = very concrete, 7 = very abstract; see Figure S4). Concreteness ratings were analyzed using 1-sample Wilcoxon signed-rank tests against the scale midpoint (4). Icons that did not differ significantly from the midpoint (*P* < 0.05) were excluded. This procedure resulted in 2 groups: concrete icons (ratings significantly below the midpoint) and abstract icons (ratings significantly above the midpoint). Inter-rater reliability of the concreteness ratings was assessed using intraclass correlation coefficients, with full results reported in the Supplementary Materials (Table S2).

The remaining 240 icons (concrete and abstract) were subsequently evaluated in an attractiveness survey (Figure S2 in Supplementary). Participants rated each icon on a 7-point scale (1 = not at all visually appealing, 7 = extremely visually appealing; see Figure S4). The instructions included the Finnish prompt “Kuinka esteettisesti miellyttäviä ikonit mielestäsi ovat?” (“How aesthetically pleasing do you perceive these icons?”). Attractiveness ratings were analyzed using the same statistical procedure to identify icons rated significantly above or below the scale midpoint, resulting in 2 distinct groups: attractive and unattractive. To ensure an equal number of icons in each condition, some icons that otherwise met the criteria were removed. All survey instructions were presented in Finnish, the participants’ native language, to ensure accurate understanding of terms and perceptual features. The Finnish versions of the instructions were translated and prepared by 2 native Finnish-speaking researchers (see Figure S1-3 in Supplementary Materials for details).

Finally, 140 icons were selected for the MEG experiment, forming a 2 × 2 factorial design with the factors concreteness (concrete vs. abstract) and attractiveness (attractive vs. unattractive), resulting in 4 experimental conditions, see Table S4. Each icon was paired with both a semantically congruent and an incongruent word prime, yielding 140 congruent and 140 incongruent trials, for a total of 280 experimental trials. In addition, 60 scrambled icons were created by spatially rearranging elements of the concrete icons to preserve low-level visual properties while removing semantic content. These served as nonsemantic visual fillers and were randomly interspersed throughout the experiment. All stimuli were presented on a gray background at a uniform size (400 × 400 pixels), resolution, and visual format.

### Procedure

**Figure 1:**
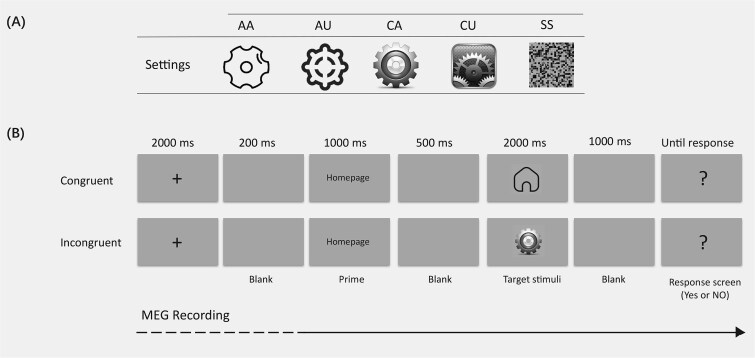
An example of stimuli and experimental procedure. (A) Example of the icons from the “settings” category, shown across 4 experimental conditions: abstract–attractive (AA), abstract–unattractive (AU), concrete–attractive (CA), and concrete–unattractive (CU). Scrambled stimuli (SS) were used as visual controls and were not included in the main experimental conditions. Each icon was presented twice across different trials to form semantically congruent or incongruent word–icon pairs. (B) Schematic of a single trial during MEG recording. Each trial began with a fixation cross (2,000 ms), followed by a prime word (1,000 ms), a brief blank (500 ms), and a target icon (2,000 ms). Participants responded only when the question mark appeared, indicating whether the prime and target were semantically congruent or incongruent.

As shown in Fig. 1B, the experiment employed a semantic judgment task. Prior to the main task, participants completed 20 practice trials to familiarize themselves with the procedure. Each trial began with a central fixation cross displayed for 2,000 ms, followed by a 200-ms blank screen. A prime word was then presented for 1,000 ms, followed by a 500-ms blank interval. Next, the target icon appeared and remained on the screen for 2,000 ms. After the target, either a question mark (“?”) or a blank screen was shown for 2,000 ms. Participants continuously performed the semantic judgment task throughout the experiment, determining on each trial whether the prime and target were semantically related (“yes”) or unrelated (“no”). A button-press response was made only when a question mark appeared after the target. The response-hand mapping was counterbalanced across participants. The question mark was randomly presented on 10% of the trials. The next trial began immediately after the response. Visual stimuli were presented using Presentation (version 2022). Prime words were displayed in Arial font, and all stimuli were projected onto a screen positioned approximately 1 m from the participants’ eyes. The prime word stimuli subtended a horizontal visual angle ranging from 3°27′ to 7°26′, while the icon stimuli subtended an angle of 2°17′ to 4°58′.

Following the MEG session, participants completed a familiarity rating survey (see Figure S3 and S4) to account for potential individual differences in prior exposure to the icon stimuli.

### Data acquisition

MEG data were recorded with the 306-channel Elekta Neuromag TRIUX system (MEGIN OY, Helsinki, Finland) in a magnetically shielded room located at the Center for Interdisciplinary Brain Research, University of Jyväskylä. Data were band-pass filtered online at 0.1 to 330 Hz and sampled at 1 kHz. Five head position indicator (HPI) coils were used to track the head position inside the MEG helmet continuously. Three of the HPI coils were placed on the participant’s forehead and 1 behind each ear. Before the MEG experiment, the position of 3 anatomic landmarks (nasion, left, and right preauricular points), the 5 HPI coils, and the head shape (>100 points evenly distributed over the scalp) were digitized using the Polhemus Isotrak digital tracker system (Polhemus, Colchester, VT, United States). Electrooculography (EOG) was recorded to monitor eye movements; 2 vertical electrodes were positioned above and below the participant’s eye to record vertical movements, and 2 horizontal electrodes were placed at the outer canthi of both eyes to capture horizontal movements, and 1 additional ground electrode was attached to the collarbone. The MEG gantry was in a 68° upright position during the recording with participants sitting comfortably in a chair. The behavioral data were collected via keypad with 2 response buttons during MEG.

### Data Preprocessing

MEG data were first preprocessed using MaxFilter (Taulu and Simola 2006) to remove external interference and compensate for head movements during recording. Spatiotemporal signal space separation with head movement compensation was applied in MaxFilter. Bad channels were identified manually and reconstructed within MaxFilter. Subsequent analysis of the MEG data was performed using minimum norm estimates (MNE)-Python (Gramfort et al. 2013). The data were first low-pass filtered at 40 Hz using a zero-phase finite impulse response filter with a Hamming window. Independent component analysis (ICA) was then performed using the FastICA algorithm (Hyvärinen 1999) to identify and remove ocular (eg blinks and saccades) and cardiac artifacts. Artifact-related ICA components were identified based on their time course, topography, and correlation with EOG and electrocardiogram (ECG) signals, and were excluded from the data prior to further analysis.

Trials were epoched from −200 to 1,000 ms relative to stimulus onset. Bad epochs were automatically rejected based on peak-to-peak amplitude thresholds of 1500 fT/cm for gradiometers and 5,000 fT for magnetometers. Additional visual inspection was performed to ensure data quality, and trials containing any residual artifacts were manually marked and excluded from subsequent analyses. Baseline correction was applied by subtracting the mean amplitude during the 200 ms prestimulus period from each time point in the epoch for each channel. ERF responses were computed by averaging the artifact-free trials separately for each of the 4 experimental conditions.

As individual magnetic resonance images (MRIs) were not available, the standard Freesurfer average brain template (“fsaverage”) was used. This template was uniformly scaled and coregistered to each participant’s digitized head shape using the automated procedure described by Houck and Claus (2020). The forward model was based on a source space generated with the “ico4” option, resulting in approximately 2,562 source locations per hemisphere with an average spacing of 6.2 mm. A single-layer boundary element model was created under the assumption of homogeneous conductivity within the intracranial volume. The noise covariance matrix was estimated from the prestimulus baseline period (−200 to 0 ms). For source analysis of the ERF responses, MNE were computed using dynamic statistical parametric mapping (Dale et al. 2000) for noise normalization. The inverse operator was constructed with free source orientations (loose = 1.0) and depth weighting (*P* = 0.8; Hämäläinen and Ilmoniemi 1994; Lin et al. 2006). For the final source estimates, only the orientation normal to the cortical surface was retained (pick_ori = “normal”) to enhance spatial specificity.

To further disentangle the neural representational structure underlying icon perception, we performed time-resolved, source-level RSA on single-trial MEG source estimates (Kriegeskorte et al. 2008) separately for each participant. Region of interest (ROI)-wise neural dissimilarity matrices were compared with 11 model RDMs, including participant-specific familiarity and attractiveness ratings, as well as concreteness, icon meaning, congruency, and low-level visual features. Familiarity was modeled along 2 theoretically motivated dimensions (Isherwood et al. 2007; Shen et al. 2018): style familiarity (1 to 7 scale), reflecting how frequently participants encountered the icon’s visual style in interfaces, serving as an index of accumulated experience with similar functional conventions; and item familiarity (0/1), indicating whether participants recognized the depicted object (ie a familiar vs. unfamiliar binary judgment). Attractiveness was included as both subjective attractiveness (1 to 7 scale) and categorical attractiveness (attractive vs. unattractive; 0/1). Concreteness was likewise included as both continuous concreteness (group-mean ratings from a separate survey; 1 to 7) and categorical concreteness (concrete vs. abstract; 0/1). Survey details and rating processing are described in the Supplementary Materials.

We constructed 6 RDMs to capture icon-related properties: categorical concreteness (0/1), continuous concreteness (1 to 7), categorical attractiveness (0/1), subjective attractiveness (1 to 7), style familiarity (1 to 7), and item familiarity (0/1). In addition, 2 semantic RDMs were included: icon category (6 function-based semantic categories, coded as 0/1) and congruency (congruent vs. incongruent icon-meaning pairings, coded as 0/1). For subjective rating variables (concreteness, attractiveness, and familiarity; 1 to 7), Euclidean distance was used to compute trial-by-trial dissimilarities. For categorical variables, dissimilarity was defined as 0 when trials belonged to the same category and 1 when they differed (Vida et al. 2017; Xu et al. 2024).

To control for low-level visual similarity in the RSA, we computed 3 image-based RDMs: a Gabor RDM, derived from a bank of Gabor filters spanning 5 spatial frequencies and 6 orientations, with the mean and variance of each filter’s magnitude envelope serving as features, capturing orientation and spatial-frequency structure analogous to early visual cortex processing (Daugman 1985); a Histogram of Oriented Gradients (HOG) RDM, derived from HOG descriptors (Dalal and Triggs 2005); 8 orientations, 8 × 8 pixels per cell, 2 × 2 cells per block, capturing contour and shape information; and a pixel RDM, based on raw grayscale luminance vectors. All stimulus images were centered on a square canvas, resized to 128 × 128 pixels, converted to grayscale, and normalized to the range [0, 1] before feature extraction. For all 3 RDMs, pairwise dissimilarities were computed using correlation distance. All 3 RDMs were included alongside the theoretical RDMs in the source-level RSA, allowing low-level visual similarity to be modeled alongside the theoretical predictors; in the main analysis, partial-Spearman RSA was used to estimate each model’s unique contribution.

RDM construction and RSA were conducted using the MNE-RSA package (version 0.10; https://users.aalto.fi/∼vanvlm1/mne-rsa/) from artifact-free single-trial data across the full experiment. RSA was performed in source space using a spatiotemporal searchlight approach. The same forward model used in the ERF analysis was applied, but the inverse solution was computed at the single-trial level using apply_inverse_epochs to obtain single-trial source estimates. Source-level RSA was then computed with the aparc_sub parcellation (448 cortical ROIs) using a temporal radius of 10 ms (Khan et al. 2018). Neural RDMs were derived within each ROI and temporal searchlight patch using correlation distance between single-trial source patterns.

To assess multicollinearity among model RDMs before the main RSA, we computed the pairwise Spearman correlation matrix and rank-transformed variance inflation factors (VIFs); multicollinearity was low (mean |r| = 0.079, maximum *r* = 0.738, all VIFs <5). We performed 2 complementary analyses: a main partial-Spearman RSA using 10 model RDMs and a supplementary Spearman RSA using all 11 model RDMs. The partial analysis excluded only the categorical concreteness (0/1) RDM because it showed the strongest redundancy with the continuous concreteness model.

### Statistical analysis

To assess differences in neural responses across experimental conditions while addressing the multiple comparisons problem, cluster-based permutation tests with spatiotemporal clustering were applied to both sensor- (magnetometer channels) and source-level ERF data (Maris and Oostenveld 2007). To avoid setting an arbitrary cluster-forming threshold, we employed threshold-free cluster enhancement (TFCE) with parameters *h* = 2.0, *e* = 0.5, start = 0, and step = 0.2 (Smith and Nichols 2009).

Five analysis time windows were predefined based on the major peaks in the grand-average global field power (GFP) of the ERFs, collapsed across all conditions, and on well-established ERP/ERF component latencies from prior studies of visual object, symbol, and face processing, ensuring a condition-independent and noncircular selection of temporal intervals. Specifically, the 80 to 130 ms window (peak ~105 ms) was taken to reflect early feedforward visual encoding (P1/M100), associated with sensitivity to low-level visual features (Di Russo et al. 2002; Liu et al. 2002; Herrmann et al. 2005; Luck 2014); the 150 to 200 ms window (peak ~175 ms) captured configural and category-level processing (N170/M170), linked to structural encoding in occipitotemporal cortex (Bentin et al. 1996; Halgren et al. 2000; Rossion and Jacques 2008); the 200 to 300 ms window (peak ~250 ms) corresponded to the transition from perceptual to semantic processing (N250/N300), associated with object representation and initial semantic access (Schendan and Kutas 2002, 2003; Tanaka et al. 2006); the 300 to 600 ms window (peak ~450 ms) indexed semantic integration and memory retrieval processes (N400/LPC) (Rugg and Curran 2007; Kutas and Federmeier 2011); and the 600 to 1,000 ms window (peak ~800 ms) captured late sustained processing related to decision evaluation and elaborative recollection (Friedman 1990; Woodruff et al. 2006; Voss and Paller 2009). Importantly, statistical inference was based on TFCE-corrected cluster-based permutation tests, which identify the exact temporal extent of significant effects independently of window boundaries. For clarity, a summary of the predefined time windows is provided in Table S9.

A 2 × 2 repeated-measures analysis of variance (ANOVA) with concreteness (abstract vs. concrete) and attractiveness (attractive vs. unattractive) as within-subject factors was used in conjunction with the cluster-based permutation test to evaluate main effects and interactions while controlling for multiple comparisons. A total of 10,000 permutations was used, and the significance level was set at α = 0.05 for all tests. To further correct for multiple testing across predefined time windows, a Bonferroni correction was applied by dividing the alpha level by the number of windows, yielding a Bonferroni-corrected significance level of α = 0.01 per window. An analogous statistical framework was applied to the RSA data: partial Spearman correlation coefficients for each model RDM were first transformed to Fisher z scores and then tested against zero using 1-sample TFCE-based permutation tests in the 5 prefined time windows.

To assess whether low-level visual properties differed systematically across experimental conditions, we computed 5 objective image-level metrics for each of the 140 unique icon stimuli. The following metrics were extracted for each icon: (i) edge density, defined as the proportion of edge pixels detected by a Canny edge detector (σ = 2.0); (ii) luminance variance, computed as the variance of pixel intensities across the image; and (iii) spatial frequency energy, obtained by applying the 2D Fourier transform to each image and measuring mean log power in 3 equal-width radial frequency bands (low, mid, and high, spanning from DC to Nyquist). These metrics were chosen to capture the visual dimensions most likely to covary with concreteness: structural complexity, luminance distribution, and spectral content. For each metric, a 2 × 2 between-item ANOVA with concreteness and attractiveness as factors was performed.

To rule out differential priming effects of the preceding word, 2 additional 2 × 2 cluster-based permutation ANOVAs (TFCE, 10,000 permutations) were conducted: congruency × concreteness (collapsing across attractiveness) and congruency × attractiveness (collapsing across concreteness). Both analyses retained approximately 70 trials per cell. Tests were performed across the same 5 predefined time windows at both sensor (magnetometer) and the source levels, using the same Bonferroni-corrected significance threshold (α = 0.01).

## Results

### Behavioral performance

Participants performed the task with high accuracy on response-required trials (mean accuracy = 82.4%, SD = 9.7%; mean RT = 1003 ms, SD = 463 ms), indicating that they followed the task instructions.

### Low-level visual properties analysis of icon stimuli

Results of the 2 × 2 ANOVAs on low-level visual metrics are summarized in Table S5 and Fig. S5. Concrete icons had significantly higher edge density than abstract icons (*F*(1, 136) = 36.40, *P* < 0.001, η^2^ = 0.210). Concrete icons also showed greater low spatial-frequency energy (*F*(1, 136) = 13.69, *P* < 0.001, η^2^ = 0.091) and mid spatial-frequency energy (*F*(1, 136) = 5.36, *P* = 0.022, η^2^ = 0.036). For attractiveness, icons rated as more attractive showed higher mid (*F*(1, 136) = 5.42, *P* = 0.021, η^2^ = 0.036) and high spatial-frequency energy (*F*(1, 136) = 8.80, *P* = 0.004, η^2^ = 0.058). Luminance variance did not differ by concreteness or attractiveness (*F* < 0.01, *P* = 0.999). No concreteness × attractiveness interactions reached significance (all *P* > 0.06).

### Grand-averaged ERFs and spatiotemporal activation patterns

**Figure 2:**
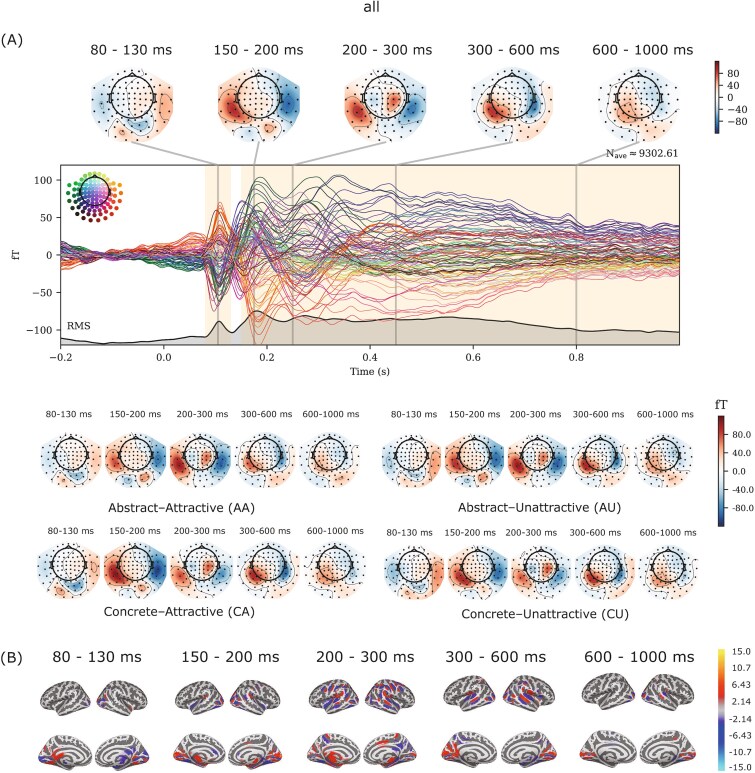
Grand average sensor- and source-level brain activity. (A) Grand-average ERF waveforms across all sensors, with topographic maps averaged over the 5 analysis time windows (80 to 130 ms, 150 to 200 ms, 200 to 300 ms, 300 to 600 ms, and 600 to 1000 ms). Maps are shown collapsed across all conditions, as well as separately for abstract vs. concrete and attractive vs. unattractive icon stimuli. (B) Distributed source activation in the left and right hemispheres across the same time windows, showing the spatiotemporal progression of cortical responses from early occipital to later, more distributed activity.

Grand average ERFs were computed by averaging baseline-corrected evoked responses across all conditions and all 35 participants at both the sensor and source levels (Fig. 2). The GFP revealed 5 major peaks around 105, 175, 250, 450, and 800 ms poststimulus onset. Source activation maps (Fig. 2B) showed early bilateral occipital activity (~100 ms), followed by progressively more distributed activation across occipital, temporal, parietal, and frontal regions in later time windows.

### Concreteness, attractiveness, and interaction effects in icon perception

#### Concreteness effect (abstract vs. concrete)

**Figure 3:**
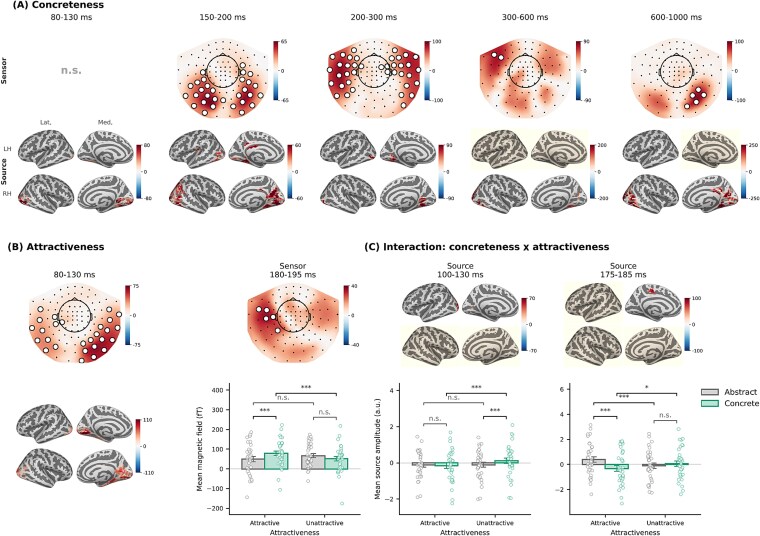
Sensor- and source-level 2 × 2 ANOVA results for concreteness (A), attractiveness (B), and their interaction (C). Panel A shows the concreteness main effect across the tested time windows, and panel B shows the significant attractiveness window. White circles mark sensors that survived the Bonferroni-corrected TFCE threshold (α = 0.01). Source maps display brain regions with significant *F*-values (TFCE-corrected) shown for lateral and medial views of the left and right hemispheres. Panel C shows the 3 significant interaction effects together with cell-mean plots extracted from the corresponding significant sensor or source masks. Interaction plots display participant-level data, mean ± SEM, and Bonferroni-corrected simple effects. n.s. = nonsignificant; TFCE = threshold-free cluster enhancement.

Concreteness significantly modulated neural responses in 4 of the 5 tested sensor-level windows and in all 5 source-level windows (all cluster-corrected *P* ≤ 0.008; Fig. 3A). At the sensor level, significant concreteness effects were observed at 150 to 190 ms (*P* < 0.001, partial η^2^ = 0.36), 210 to 300 ms (*P* < 0.001, partial η^2^ = 0.34), 375 to 390 ms (*P* = 0.008, partial η^2^ = 0.35), and 610 to 835 ms (*P* < 0.001, partial η^2^ = 0.35). The effects were primarily distributed over posterior sensors, with a more widespread distribution at later time windows. At the source level, significant concreteness effects were present at 90 to 130 ms (*P* < 0.001, partial η^2^ = 0.37), 150 to 200 ms (*P* < 0.001, partial η^2^ = 0.34), 200 to 300 ms (*P* < 0.001, partial η^2^ = 0.32), 300 to 600 ms (*P* < 0.001, partial η^2^ = 0.30), and 600 to 1,000 ms (*P* < 0.001, partial η^2^ = 0.31). The earliest effect (90 to 130 ms) was observed in bilateral occipital cortices. From 150 ms onwards, effects extended to occipitotemporal regions and later involved temporal and parietal cortices, becoming more widespread at later time windows. Across all significant windows, concrete icons elicited stronger neural responses than abstract icons. Cluster-averaged marginal mean absolute values and SEs, computed over the exact significant TFCE masks, are reported in Supplementary Table S7 (Table S7-1).

#### Attractiveness effect (attractive vs. unattractive)

Attractiveness showed a transient early main effect. A significant effect was observed at the sensor level from 95 to 130 ms (*P* < 0.001, partial η^2^ = 0.34) and at the source level from 80 to 130 ms (*P* < 0.001, partial η^2^ = 0.29; Fig. 3B) in occipital and ventral occipitotemporal regions, consistent with early visual and perceptual processing. Cluster-averaged marginal mean absolute values and SEs for the 2 attractiveness levels are reported in Table S7-2.

#### Interaction effect (concreteness × attractiveness)

A significant interaction was observed at the sensor level between 180 and 195 ms (TFCE-corrected *p* [*p*TFCE] = 0.005, partial η^2^ = 0.50; Fig. 3C). The effect was primarily distributed over left posterior sensors, with a maximum over parietal regions. Cluster-averaged cell means showed a crossover pattern (see Table S7-3). Bonferroni-corrected simple-effects analyses showed that the concreteness effect was significant only for attractive icons, *t*(34) = −4.19, *P* < 0.001, *d* = −0.71, whereas the attractiveness effect was significant only for concrete icons, *t*(34) = 4.30, *P* < 0.001, *d* = 0.73. At source level, 2 interaction windows reached significance: 100 to 130 ms (*p*TFCE = 0.002, partial η^2^ = 0.76) and 175 to 185 ms (*p*TFCE = 0.006, partial η^2^ = 0.78). The early interaction (100 to 130 ms) was localized to occipital regions. The later interaction (175 to 185 ms) was localized to left parietal cortex. Both clusters showed crossover patterns in the condition means (Table S7-3). The early source interaction reflected a concreteness effect for unattractive icons, *t*(34) = −5.11, *P* < 0.001, *d* = −0.86, and an attractiveness effect for concrete icons, *t*(34) = −7.43, *P* < 0.001, *d* = −1.26. The later source interaction reflected a concreteness effect for attractive icons, *t*(34) = 6.64, *P* < 0.001, *d* = 1.12, together with attractiveness effects for both abstract icons, *t*(34) = 5.21, *P* < 0.001, *d* = 0.88, and concrete icons, *t*(34) = −2.94, *P* = 0.023, *d* = −0.50, with opposite directions across concreteness levels.

#### Priming control (congruency interactions)

The congruency × concreteness and congruency × attractiveness interactions were not significant in any analyzed time window at either the sensor or source level (all *p*TFCE ≥0.181; Table S6). At the sensor level, a main effect of congruency emerged in the 300 to 600 ms window (*P* = 0.001), consistent with later semantic/N400-like processing, but this effect was additive and did not modulate either the concreteness or attractiveness effects (Fig. S6). The corresponding source-level congruency effect in this window did not survive TFCE correction (*P* = 0.068 for the congruency × concreteness ANOVA; *P* = 0.072 for the congruency × attractiveness ANOVA).

### Neural representations analysis of icon perception

**Figure 4:**
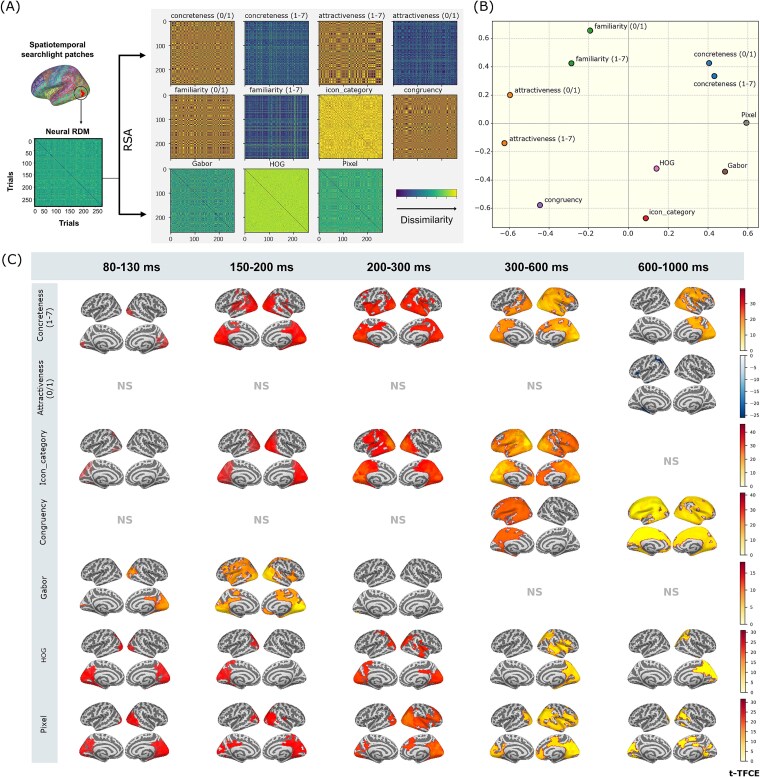
Spatiotemporal RSA of icon processing. (A) Schematic illustration of the RSA pipeline. Neural RDMs were computed for each searchlight patch across time and ROIs defined by aparc_sub parcellations and were correlated with 11 model RDMs derived from icon visual, perceptual, and semantic features. (B) Multidimensional scaling (MDS) of all model RDMs based on group-average correlations, showing that binary and continuous versions of each construct tend to cluster together, concreteness and low-level visual models are separated from attractiveness and familiarity along MDS, and icon meaning and congruency occupy distinct positions in the lower portion of the space. (C) Source-level brain maps from the partial Spearman RSA across the 5 predefined time windows (80 to 130, 150 to 200, 200 to 300, 300 to 600, and 600 to 1,000 ms). Rows correspond to model RDMs showing at least 1 significant effect, and columns correspond to time windows; cells labeled n.s. were not significant. Maps show ROIs surviving the Bonferroni-corrected TFCE threshold (α = 0.01), rendered in lateral and medial views of both hemispheres.

The RSA analyses revealed a clear spatiotemporal progression in the representational geometry underlying icon processing (Fig. 4 and Fig. S7). In the zero-order Spearman RSA (Fig. S7), both concreteness models (0/1 and 1 to 7), icon category, and all 3 low-level visual RDMs were significant across all 5 time windows. By contrast, congruency showed a distinctly later profile, reaching significance only at 300 to 600 and 600 to 1,000 ms. Effects of attractiveness and familiarity were more limited: only categorical attractiveness (0/1) was significant at 80 to 130 and 150 to 200 ms and showed an additional late effect at 600 to 1,000 ms, whereas continuous attractiveness (1 to 7) did not reach significance in any window. Familiarity effects were similarly sparse; item familiarity (0/1) was significant only at 300 to 600 ms and style familiarity (1 to 7) was only at 200 to 300 and 600 to 1,000 ms.

The partial-Spearman RSA (Fig. 4), which isolated the unique contribution of each model after controlling for all others, yielded a more selective pattern. As the categorical concreteness (0/1) model was excluded from the partial analysis due to redundancy, the continuous concreteness (1 to 7) model was examined here. It retained significance across all 5 time windows, emerging first in bilateral primary visual cortex (80 to 130 ms) and progressively extending to temporal, parietal, and frontal regions at later latencies, confirming a robust and spatiotemporally sustained unique contribution to the neural representational structure of icons. Icon category also retained unique effects from 80 to 600 ms with a similar spatial progression from early visual to more distributed cortical regions, although its effect in the 600 to 1,000 ms window was no longer significant in the partial RSA. Congruency showed a uniquely late profile, reaching significance only at 300 to 600 and 600 to 1,000 ms, with effects focused in bilateral temporal, parietal, and frontal regions.

Among the low-level visual models, pixel and HOG similarity explained unique variance across all 5 time windows, whereas Gabor similarity was restricted to the 3 earliest windows (80 to 300 ms), with its later effects not reaching significance in the partial RSA. All 3 visual models showed relatively focal effects in bilateral occipital cortex during the first 300 ms, extending to temporal and frontal regions at later latencies. In contrast, neither familiarity model (item familiarity, 0/1 and style familiarity, 1 to 7) reached significance in any time window after controlling for the other predictors. The early categorical attractiveness effects in bilateral occipital cortex observed in the zero-order analysis were also no longer significant in the partial RSA analysis, leaving only a late effect at 600 to 1,000 ms. Cluster-averaged mean correlation coefficients, SDs, and Cohen’s *d* values for all significant effects in both the zero-order (Table S8-1) and partial-Spearman RSA (Table S8-2) are reported in Supplementary Table S8.

## Discussion

The present study examined how concreteness and attractiveness shape the spatiotemporal dynamics of icon perception. Using MEG in combination with source imaging and RSA, we found that these 2 icon features influence neural processing in qualitatively different ways. Concreteness exerted a robust and sustained influence from as early as 80 ms and persisted through 1,000 ms, with activation propagating from bilateral occipital cortices to temporal and parietal regions. In contrast, attractiveness gave rise to early but transient neural differentiation within the first 130 ms, an effect that did not propagate into later processing stages and was largely attributable to low-level visual properties rather than reflecting an independent esthetic contribution. A significant concreteness × attractiveness interaction further revealed that these 2 dimensions do not operate entirely independently, with their joint influence manifesting at both early occipital and later parietal stages of processing, though in the absence of corresponding behavioral differences, this interaction is interpreted in an exploratory manner. The partial RSA revealed dissociable representational dynamics, with concreteness showing sustained correspondence with neural representational structure across all processing stages, while attractiveness, familiarity, and icon category each contributed in temporally distinct and more limited ways.

The concreteness effect was evident across a broad temporal range, emerging early and persisting into later stages of processing, indicating that concreteness serves as a stable organizing dimension in icon perception. The earliest concreteness effect emerged approximately 80 to 130 ms after stimulus onset and was localized to the bilateral occipital cortices. This latency is typically associated with early visual perceptual encoding, consistent with the view that the visual system is sensitive to differences in icon concreteness at an initial stage of processing (Grill-Spector and Kanwisher 2005; Kuefner et al. 2010). Compared with abstract icons, concrete icons elicited stronger or more readily discriminable neural responses during this early window, supporting the hypothesis that concreteness modulates early perceptual encoding, potentially due to richer and more structured visual information (McDougall et al. 1999; Collaud et al. 2022). Although low-level visual properties such as color, size, and pixel format were carefully controlled across stimuli, concrete icons inevitably contained greater structural detail and visual complexity due to their representational nature. This interpretation is further supported by our analysis of low-level visual properties (see Table S5 and Fig. S5). Concrete icons exhibited higher edge density and greater low- and mid-spatial frequency energy than abstract icons, indicating systematic differences in visual structure. From approximately 150 ms onward, the concreteness effect became more widespread, extending from occipital regions into lateral temporal and parietal cortices. This posterior-to-lateral progression aligns with hierarchical models of visual processing, in which early perceptual distinctions propagate along the ventral visual pathway as representations become increasingly refined and differentiated (Grill-Spector et al. 2001). Prior neuroimaging studies have shown that icons recruit occipitotemporal regions associated with object recognition and visual concept formation (Shin et al. 2008; Huang et al. 2015). The present findings converge with and extend this spatial pattern by demonstrating that concreteness-related effects initially arise in the occipital cortex and subsequently extend into lateral temporal and parietal regions, further characterizing the temporal evolution of this progression.

The concreteness effect remained detectable at later latencies (approximately 300 to 600 ms) and in some regions extended beyond this window, indicating a sustained influence of concreteness on neural dynamics (Adorni and Proverbio 2012; Kiefer and Pulvermüller 2012). This sustained activity is consistent with classical accounts of conceptual representation, such as Dual Coding Theory (Clark and Paivio 1991; Paivio 1991), which posits that concrete concepts are supported by both verbal and imagery-based representational systems, whereas abstract concepts rely more heavily on linguistic codes alone. Concrete concepts have been shown to engage distributed multimodal representations grounded in perceptual and sensorimotor experience, giving rise to more sustained and widespread cortical activation than abstract concepts (Binder and Desai 2011). Accordingly, concrete icons may continue to engage imagery-related and perceptually grounded conceptual representations beyond early sensory encoding, giving rise to prolonged neural differentiation at later stages. Importantly, priming control analyses indicate that these effects cannot be attributed to differential semantic priming. Although a main effect of congruency was observed in the 300 to 600 ms window, it did not interact with either concreteness or attractiveness. This pattern is consistent with a temporally distinct stage of context-dependent processing and does not account for the feature-related effects reported here.

The interpretation of these findings is further informed by low-level visual property analyses and RSA. Analysis of objective image-level metrics confirmed that concrete icons exhibited significantly higher edge density and greater low- and mid-spatial frequency energy than abstract icons, indicating systematic differences in visual structure that may contribute to the early neural differentiation observed. However, the partial RSA results demonstrated that concreteness retained a significant and unique contribution to neural representational structure across all 5 time windows, emerging first in the bilateral primary visual cortex at 80 to 130 ms and progressively extending to temporal, parietal, and frontal regions at later latencies, even after controlling for pixel, HOG, and Gabor similarity and other icon-related features (Fig. 4). This indicates that the observed neural differences in concreteness cannot be fully explained by low-level visual complexity or spatial frequency content alone, but instead reflect higher-level representational distinctions associated with icon meaning that are engaged remarkably early and sustained throughout processing.

In contrast to the sustained and widespread influence of concreteness, attractiveness gave rise to a transient early main effect, with attractive icons eliciting stronger neural responses than unattractive icons within a narrow temporal window from 80 to 130 ms. At the sensor level, this effect was observed from 95 to 130 ms over posterior sensors, with the corresponding source-level effect localized to bilateral occipital and ventral occipitotemporal regions. The early latency and occipital localization of this effect are consistent with rapid, automatic sensitivity to perceptual properties of icons at an initial stage of visual processing, suggestive of enhanced perceptual salience rather than higher-order esthetic appraisal. This early modulation may reflect automatic attentional capture by visually appealing stimuli, consistent with evidence that perceptually salient features can orient attention involuntarily at early processing stages, prior to conscious evaluation (Batty and Taylor 2003; Vuilleumier and Pourtois 2007). Notably, the attractiveness effect did not propagate into later processing stages, and no activation emerged in temporal–parietal or prefrontal regions associated with evaluative or conceptual processing. This pattern contrasts with classical neuroesthetic findings, in which esthetic judgments of artworks reliably engage medial prefrontal and orbitofrontal cortices at later stages, reflecting affective valuation and conscious appraisal (Cela-Conde et al. 2004; Kawabata and Zeki 2004; Ishizu and Zeki 2011). We argue that this divergence arises from both stimulus type and task demands. Unlike artworks, icons are functional graphical symbols optimized for rapid recognition rather than esthetic contemplation. Moreover, participants performed a semantic task rather than an explicit evaluative judgment, which likely suppressed late-stage esthetic appraisal, consistent with prior evidence that hedonic neural effects extend into later components only when evaluative processing is task-relevant (Höfel and Jacobsen 2007; Munar et al. 2011).

The interpretation of this early attractiveness effect is further informed by low-level visual property analyses and RSA. Attractive icons exhibited significantly higher mid- and high-spatial frequency energy than unattractive icons, indicating systematic differences in low-level visual structure that covary with attractiveness ratings. Critically, the partial RSA demonstrated that the early attractiveness effect observed in the zero-order analysis did not survive after controlling for other predictors, including low-level visual feature models, suggesting that the early neural differentiation associated with attractiveness is largely attributable to variance shared with low-level visual properties rather than reflecting an independent contribution of esthetic quality per se. Only a weak and late effect at 600 to 1,000 ms survived in the partial RSA, which may reflect a limited evaluative or reflective processing stage at later latencies (Leder et al. 2004; Chatterjee and Vartanian 2014).

In addition to this account, alternative explanations should be considered. Participants provided subjective attractiveness ratings that capture individual variability in esthetic evaluation, but trait-level esthetic sensitivity was not assessed. It is therefore possible that frontal engagement related to esthetic processing may emerge more strongly in individuals with higher esthetic sensitivity. Moreover, MEG has lower sensitivity to deep and radial frontal sources, which may have reduced our ability to detect subtle frontal activity, although frontal effects were observed in the RSA analyses at later latencies. Taken together, these considerations suggest that the absence of late frontal effects for attractiveness should be interpreted with caution. Overall, these findings suggest that under task conditions emphasizing semantic processing, icon attractiveness operates primarily as a low-level visual property that boosts early perceptual salience, rather than as a dimension of esthetic evaluation recruiting higher-order appraisal networks.

The significant concreteness × attractiveness interaction revealed that these 2 dimensions do not operate independently in shaping the neural processing of icons, though the nature of their interaction differed markedly across processing stages (Fig. 3C). A crossover pattern was observed across interaction windows, indicating that the influence of 1 feature depends on the level of the other rather than reflecting a simple additive effect, with the direction and magnitude of both concreteness and attractiveness effects varying across conditions.

The early source-level interaction (100 to 130 ms), localized to occipital regions, is most parsimoniously interpreted as reflecting the modulation of early perceptual encoding by the combined influence of low-level visual structure. Concrete icons possess richer and more structured visual properties, rendering attractiveness-related perceptual differences more discriminable at early stages of visual analysis, whereas abstract icons lack sufficient visual structure to support such early differentiation. As such, this early interaction likely reflects the joint influence of low-level visual features rather than a genuinely higher-order integration of semantic and esthetic dimensions.

The later interaction (175 to 195 ms), localized to left parietal cortex, presented a qualitatively different pattern. The left parietal localization and intermediate latency are consistent with higher-order perceptual and attentional integration processes unlikely to be fully accounted for by low-level visual structure alone. One possible interpretation is that when icons provide limited visual-conceptual information, as in the case of abstract icons, perceptual processing may rely more strongly on additional cues such as visual salience or evaluative properties. More generally, the crossover pattern suggests that the perceptual influence of attractiveness is modulated by the availability of visual-conceptual information: when icons are concrete and already provide rich structural cues, attractiveness may contribute less additional modulation of early perceptual processing, whereas for abstract icons with fewer direct visual cues, attractiveness may play a relatively greater role in guiding early perceptual encoding, consistent with behavioral evidence that the association between esthetic appeal and concreteness is weak and context-dependent (Zheng et al. 2026).

The observed interaction is also broadly compatible with accounts linking esthetic evaluation to perceptual fluency, in the sense that early neural modulation may reflect differences in processing efficiency (Reber et al. 2004; Preßler, 2023). However, such accounts do not directly explain the observed crossover pattern, and this interpretation remains exploratory. Importantly, the interaction was transient and confined to early processing stages, with no sustained effects observed at later latencies. Together with the absence of corresponding behavioral interactions, this pattern is best interpreted as reflecting a stimulus-dependent modulation of perceptual encoding rather than a stable interaction at the level of semantic access or decision-making.

The partial RSA analyses further revealed dissociable representational contributions from icon category, congruency, and familiarity, providing further insight into the dimensions that organize neural icon representations beyond concreteness and attractiveness (Fig. 4). Icon category showed a consistent but temporally constrained contribution to neural representational structure (80 to 600 ms), indicating that category-level information supports the early organization of icon representations. This likely reflects the fact that category membership is closely tied to shared visual and functional features, allowing it to be accessed without requiring extensive contextual processing. Its reduced contribution at later stages suggests that, once more specific interpretations are established, broader categorical distinctions become less informative for ongoing processing. In contrast, congruency showed a selectively late contribution, reaching significance only from 300 ms onward in bilateral temporal, parietal, and frontal regions. This pattern indicates that congruency reflects a distinct and later stage of processing. Because congruency depends on the alignment between the prime and the icon, it requires the integration of external contextual information with the visual input, making it inherently a higher-level, context-dependent process that emerges after the initial representation of the icon has been formed. This is consistent with the priming control analyses in the ERF data, which similarly showed that congruency effects emerged only at later latencies and did not modulate the concreteness or attractiveness effects. Altogether, this pattern suggests a transition from stimulus-driven representational organization to context-dependent semantic evaluation.

Familiarity did not show a reliable independent contribution to neural representational structure in the partial RSA, indicating that it does not constitute a primary organizing dimension once other factors are controlled. This finding is important as it rules out differential familiarity as an alternative explanation for the concreteness and attractiveness effects reported here. The stronger effects observed for style familiarity relative to item familiarity in the zero-order RSA analysis (see Fig. S7) suggest that graded familiarity distinctions may align more closely with neural representational structure than a simple familiar versus unfamiliar categorization, potentially because style familiarity captures shared regularities in visual form across icons, whereas item familiarity is more idiosyncratic (Yonelinas, 2002). Nevertheless, neither familiarity measure survived partial analysis, indicating that familiarity-related variance is largely shared with concreteness and icon meaning rather than reflecting an independent contribution to representational geometry.

Collectively, these RSA findings reveal a clear temporal dissociation among icon features. Concreteness acts as a dominant and sustained organizing dimension across all processing stages, whereas attractiveness exerts only a weak and temporally limited independent influence. Category-level information contributes to early and intermediate representational organization, congruency reflects late-stage context-dependent semantic integration, and familiarity does not provide an independent contribution beyond variance shared with other factors. Overall, these findings indicate that neural representations of icons are primarily structured by perceptual form and semantic content, with esthetic and familiarity-related dimensions playing secondary and largely derivative roles.

There are also several limitations of the present study that are worth noting. First, source reconstruction relied on a template brain model due to the absence of individual MRI data, which may have reduced spatial localization accuracy. Future studies could improve source precision by incorporating participant-specific anatomical scans. Second, several icon-related dimensions were assessed using subjective ratings. In particular, perceived attractiveness is highly susceptible to individual and cultural differences, which may affect the consistency of esthetic evaluations across participants. Although all stimuli were presented in grayscale to minimize potential confounds arising from color, color itself is an important contributor to esthetic perception and should be systematically examined in future research. Third, the MEG paradigm was optimized for neural measurement, with only ~10% of trials requiring a behavioral response (a congruency judgment serving as an attention check). The resulting ~8 trials per condition cell per participant are insufficient for reliable brain–behavior correlation analyses. Future studies employing tasks with higher response rates and behavioral measures more directly tied to the experimental factors (eg semantic categorization or concreteness rating tasks) would be better positioned to examine whether individual differences in neural effects predict behavioral performance. Finally, this study focused on perceptual features of isolated icons and did not address how meaning is constructed or integrated within contextualized settings. Future research should therefore investigate how perceptual attributes such as concreteness and attractiveness influence semantic integration during icon perception in more ecologically valid contexts.

## Conclusion

In conclusion, the present study provides a comprehensive characterization of the spatiotemporal dynamics through which the brain processes icons, demonstrating that perceptual and esthetic features contribute in qualitatively distinct ways to neural processing and representational organization. Our findings show that concreteness shapes neural activity from the earliest stages of visual encoding and continues to exert sustained influence across multiple subsequent processing stages, with converging evidence from spatiotemporal RSA. By contrast, attractiveness and familiarity exert more limited and temporally restricted influences. Attractiveness primarily modulates early perceptual processing through low-level visual properties and does not constitute an independent organizing dimension of neural representations, while familiarity shows only modest effects at later stages that do not survive control for other factors. Together, these results indicate that icon processing is fundamentally structured by perceptual-conceptual content, with esthetic attributes contributing to early sensory modulation rather than shaping higher-order representational organization.

These findings carry broader implications for our understanding of visual symbol processing and icon design. By establishing that concreteness, a dimension closely tied to the referential and semantic content of icons, dominates neural representational structure from early perceptual stages onward, the present work highlights the primacy of meaning-based processing in how the brain encodes functional visual symbols. This stands in contrast to the more peripheral role of esthetic appeal, suggesting that effective icon design may rely more critically on semantic clarity than on visual attractiveness. More broadly, this work advances the neuroscientific understanding of icon perception and provides a methodological and theoretical foundation for future research on visual communication, symbolic meaning construction, and the neural bases of human–computer interaction.

## Supplementary Material

Supplementary_materials_bhag075

## Data Availability

The experimental paradigm, data preprocessing, and data analysis scripts are freely available at the following Github repository: https://github.com/weiyongxu/iconmind-analysis.
