# Supplementary material for: Cortical dynamics of icon perception: effects of concreteness and attractiveness
Source: Cereb Cortex. 2026 Jun 22;36(6):bhag075. doi: 10.1093/cercor/bhag075 (PMC13286001; doi:10.1093/cercor/bhag075)
Supplement: Supplementary_materials_bhag075 [file supplementary_materials_bhag075.docx]

# **SUPPLEMENTARY MATERIAL**

**Cortical Dynamics of Icon Perception: Effects of Concreteness and Attractiveness**

Jiaqi Zheng*^1, 2,4^, Weiyong Xu ^3,4^, Johanna Silvennoinen ^1^, Fengyu Cong ^1,2,5,6^, Tiina Parviainen ^3,4^, Tuomo Kujala ^1^

1. Faculty of Information Technology, University of Jyväskylä, P.O. Box 35, FI-40014, Jyväskylä, Finland

2. School of Biomedical Engineering, Faculty of Medicine, Dalian University of Technology, Dalian 116024, Liaoning Province, China

3. Department of Psychology, University of Jyväskylä, Mattilanniemi 6, 40014, Jyväskylä, Finland

4. Centre for Interdisciplinary Brain Research, University of Jyväskylä, Mattilanniemi 6, 40014, Jyväskylä, Finland

5. Key Laboratory of Social Computing and Cognitive Intelligence, Dalian University of Technology, Ministry of Education, China

6. School of Software Engineering, Dalian University, Dalian, China

***Corresponding author:**

Jiaqi Zheng

Faculty of Information Technology, University of Jyväskylä

Mattilanniemi 2, 40100, Jyväskylä, Finland.

Email: [jzheng@jyu.fi](mailto:jzheng@jyu.fi)

### **Concreteness Rating Survey (7-point Likert scale)**

In the present study, *icon concreteness* was operationalized as a perceptual feature, referring to the extent to which an icon’s visual form resembles a real-world object or physical entity. This operationalization differs from conventional linguistic or conceptual definitions of concreteness in the cognitive literature and instead emphasizes perceptual resemblance in icon design [(McDougall et al., 1999 ; Collaud et al. 2022)](https://paperpile.com/c/1Obtfv/Z6zh+I9zl).

To promote consistent interpretation of the rating scale, participants were presented with an example image illustrating a continuum of visual concreteness, ranging from very concrete icon representations to very abstract representations. The slider scale served as a visual anchor to guide participants’ judgments of how concrete or abstract an icon appeared. All instructions and labels were presented in Finnish, the participants’ native language. The Finnish text explained concreteness and abstractness in terms of visual resemblance to real-world objects and the use of detailed versus simplified visual elements (see Figure S1).

Participants rated each icon on a 7-point Likert scale ranging from 1 = very concrete (*Hyvin konkreettinen*), through 4 = neutral, to 7 = very abstract (*Hyvin abstrakti*), with example images provided to illustrate the full range of the scale. The rating prompt presented in Finnish was: *“Kuinka abstraktina tai konkreettisena pidät näkemääsi ikonia?”*  (English: “How abstract or concrete do you perceive this icon to be?”).

Because the icon set was extensive, the concreteness rating task was divided into two separate survey parts. Inter-rater reliability was assessed using intraclass correlation coefficients (ICCs), which indicated excellent agreement across both subsets (ICC range = .86–.95, all *p* < .001; see Table S2).

### **Attractiveness Rating Survey (7-point Likert scale)**

Attractiveness was defined as the subjective aesthetic appeal of an icon, reflecting individual visual preference. Participants rated each icon’s visual attractiveness on a 7-point Likert scale ranging from 1 = not at all visually appealing (*esteettisesti hyvin epämiellyttävä*), through 4 = neutral (*neutraali*), to 7 = extremely visually appealing (*esteettisesti hyvin miellyttävä*).

The rating prompt presented in Finnish was: *“Kuinka esteettisesti miellyttävä tämä ikoni mielestäsi on?”* (English: “How aesthetically pleasing do you perceive this icon?”). All instructions were presented in Finnish, the participants’ native language (see Figure S2).

The same participant pool was subsequently invited to take part in the MEG experiment. To minimize short-term familiarity effects, the MEG session was scheduled approximately 1–2 months after completion of the attractiveness survey.

### **Familiarity Rating Survey (7-point Likert scale and binary judgment)**

To account for individual differences in prior exposure to the icons and their design styles, familiarity was assessed along two complementary dimensions [(Shen et al. 2018; Isherwood et al. 2007](https://paperpile.com/c/1Obtfv/4CnH+Yeos); see Figure S3[)](https://paperpile.com/c/1Obtfv/4CnH).

**(1) Style familiarity (or Icon-style familiarity; 1-7 scale)** refers to the extent to which users are familiar with the functional meanings typically conveyed by icons belonging to a given style category. This form of familiarity reflects accumulated experience with icons serving similar functions, such that higher usage frequency or repeated encounters facilitate faster recognition. SF therefore captures experience-based familiarity rooted in users’ prior interaction with icons of a particular functional style. Participants rated style familiarity on a 7-point Likert scale (1 = not at all familiar / *Ei lainkaan tuttuna*, 4 = neutral / *neutraali*, 7 = very familiar / *Hyvin tuttuna*).

**(2) Item familiarity (binary judgement 0/1) r**efers to familiarity with the visual objects or elements depicted in an icon, independent of its intended function. For example, a user may recognize a graphical element (e.g., a book) as visually familiar even if they are unsure about the icon’s functional meaning. In this case, the icon’s visual form is familiar whereas its function may not be. Icon-itself familiarity was assessed using a binary judgment (1 = Yes, familiar; 0 = No, unfamiliar).

In summary, the three rating surveys provided complementary assessments of icon perceptual features, including concreteness, aesthetic attractiveness, and familiarity. Examples of the rating tasks are shown in Figure S4.

**
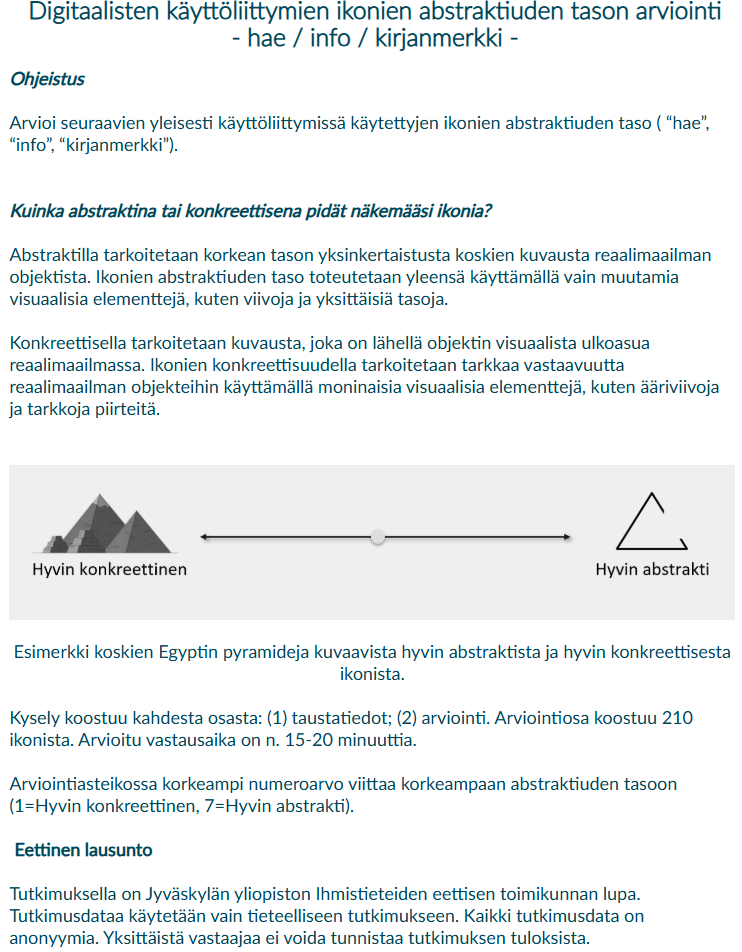
**

**Figure S1.** Instructions for the Concreteness Rating Survey.


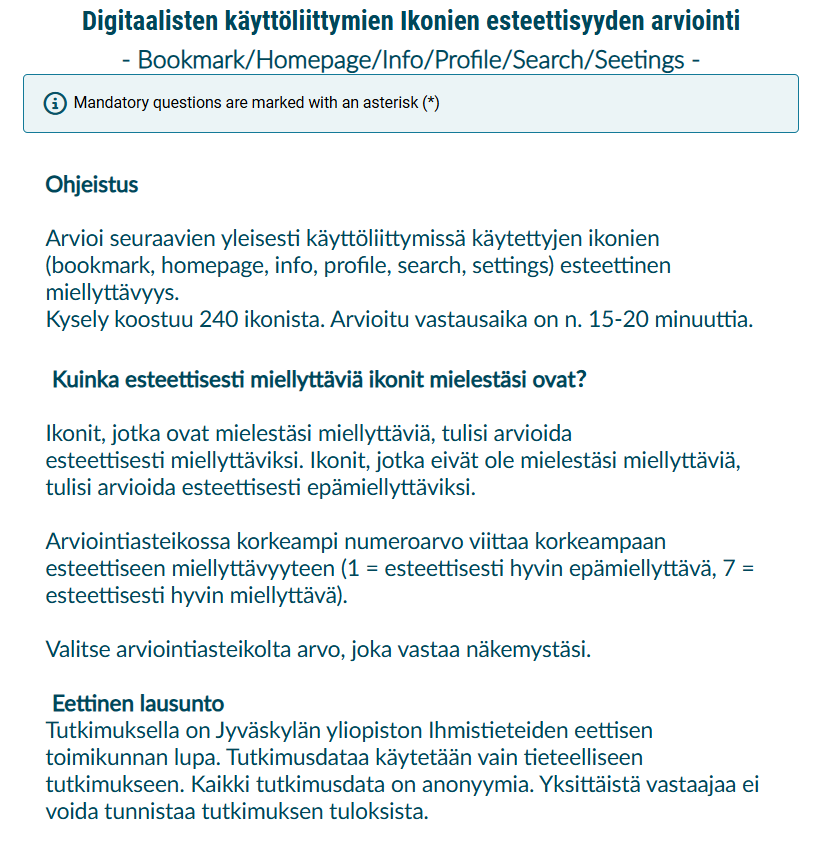


**Figure S2.** Instructions for the Attractiveness Rating Survey.


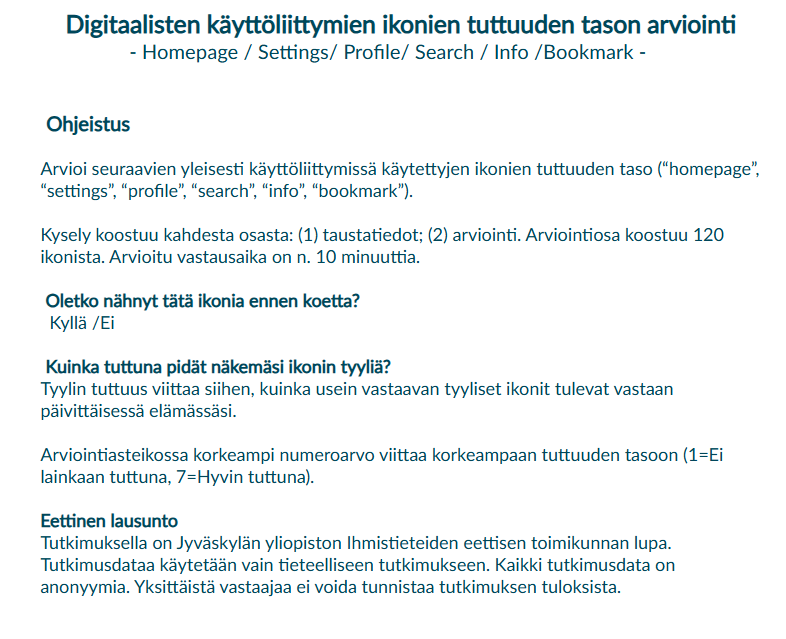


**Figure S3.** Instructions for the Familiarity Rating Survey.


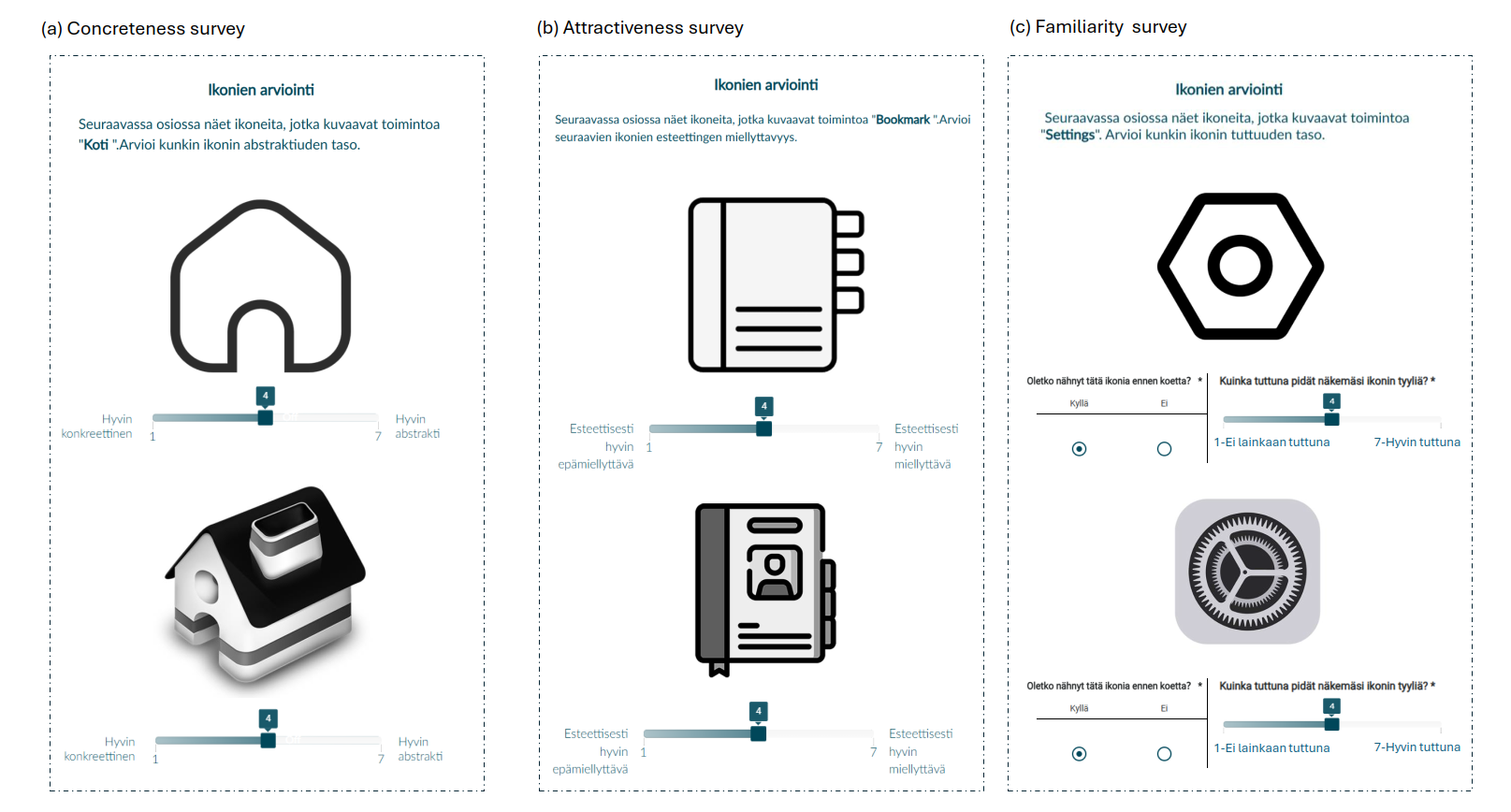


**Figure S4.** Example rating tasks used in the (a) concreteness, (b) attractiveness, and (c) familiarity surveys.

**Table S1**. Demographics of Three behavioural survey

| **Measure (Survey)** | | ***Participants*** | **Mean Age (SD)** |
| --- | --- | --- | --- |
| Concreteness | Part I | N=36 (14 males, 17 females, 2 non-binary, and 3 who preferred not to disclose) | 27.3 (8.6) |
|  | Part II | *N*=39 (18 females, 18 males, 1 non-binary, and 2 who preferred not to disclose) | 29.8 (9.5) |
| Attractiveness | | *N*=64 (35 females, 29 males) | 27.6 (6.6) |
| Familiarity | | N=35 (14 females, 1 preferred not to disclose) | 27.96 (6.91) |

**Table S2**. Inter-rater Reliability of Concreteness Ratings (ICC)

| **Survey** | **Concreteness** | ***N*** | **ICC (Average)** | **95% CI (Average)** | ***F* (df₁, df₂)** | ***P*** |
| --- | --- | --- | --- | --- | --- | --- |
| Part I | Abstract | 39 | .953 | [.926, .974] | 21.411 (29, 1102) | < .001 |
|  | Concrete | 39 | .922 | [.876, .957] | 12.861 (29, 1102) | < .001 |
| Part II | Abstract | 36 | .882 | [.810, .936] | 8.444 (28, 980) | < .001 |
|  | Concrete | 36 | .860 | [.778, .923] | 7.160 (29, 1015) | < .001 |

**Table S3**. Summary of variables collected from behavioural surveys.

| **Measure (Survey)** | **Variable Type** | **Scale/Questions** |
| --- | --- | --- |
| Concreteness | Ordinal rating  (7-point Likert) | * To what extent do you consider the icon to be abstract?  (1 = very concrete, 4= neutral, 7 = very abstract) |
| Attractiveness | Ordinal rating  (7-point Likert) | * To what extent do you find the icons aesthetically pleasing?  (1 = High attractive, 4= neutral, 7 = low attractive) |
| Familiarity (style) | Ordinal rating  (7-point Likert) | * To what extent do you find the icon's style familiar?  (1 = very familiar, 4= neutral, 7 = very unfamiliar) |
| Familiarity (item) | Binary judgment  (0/1) | * Were you familiar with this icon before the experiment?  1 = familiar, 0 = unfamiliar |

**Table S4.** Final icon counts per condition across six function-based semantic categories, balanced across concreteness × attractiveness


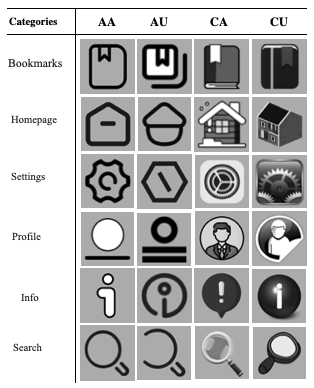


**Table S5.** Two-way ANOVA results for low-level visual metrics of icon stimuli.

| Metric | F_Conc_ | p_Conc_ | η²_Conc_ | F_Attr_ | p_Attr_ | η²_Attr_ | F_Int_ | p_Int_ | η²_Int_ |
| --- | --- | --- | --- | --- | --- | --- | --- | --- | --- |
| Edge density | 36.40 | < .001 | .210 | 0.96 | .330 | .006 | 0.02 | .886 | < .001 |
| Luminance variance | < 0.01 | .999 | < .001 | 0.15 | .695 | .001 | 2.66 | .105 | .019 |
| Low SF energy | 13.69 | < .001 | .091 | 0.46 | .497 | .003 | 0.97 | .326 | .006 |
| Mid SF energy | 5.36 | .022 | .036 | 5.42 | .021 | .036 | 3.55 | .062 | .024 |
| High SF energy | 2.19 | .142 | .015 | 8.80 | .004 | .058 | 3.56 | .061 | .024 |

*Note.* All ANOVAs used *df* = (1, 136), *n* = 35 icons per cell. Bold values indicate *p* < .05. Conc = Concreteness, Attr = Attractiveness, Int = Interaction. η² = eta-squared.


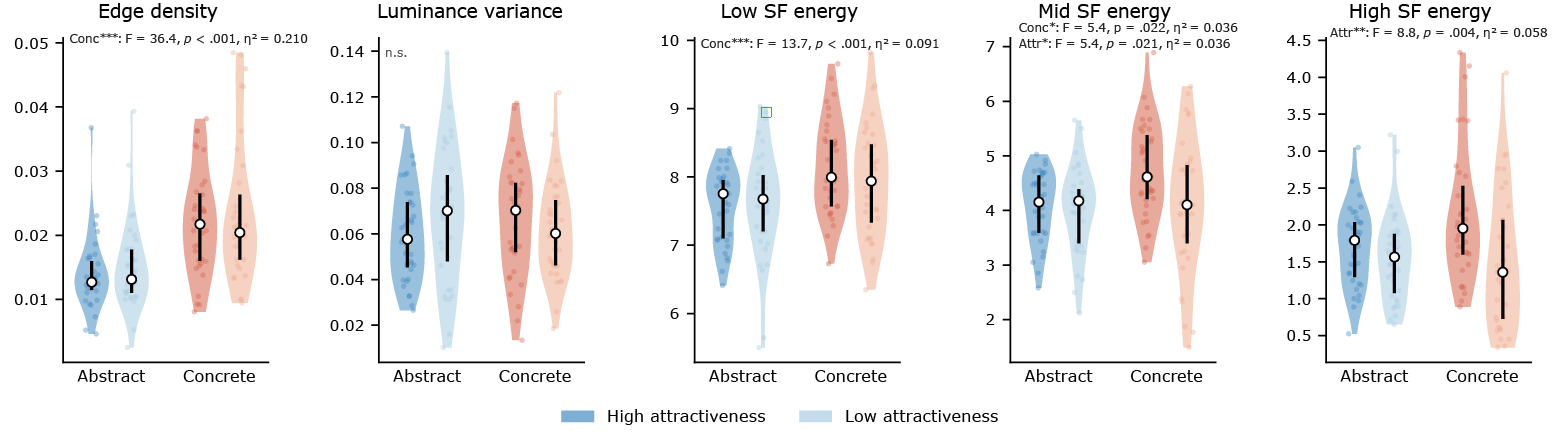


**Figure S5.** Distribution of low-level visual metrics across the four experimental conditions (Abstract/Concrete × High/Low Attractiveness). Violin plots show kernel density estimates; white circles and bars indicate medians and interquartile ranges; individual icons (*N* = 35 per condition) are overlaid with jitter. Significant ANOVA effects are annotated in each panel. *** *p* < .001, ** *p* < .01, * *p* < .05.

**Table S6.** Priming control ANOVA results — Interaction effects (Congruency × Factor). All values are TFCE-corrected *p*-values (1,024 permutations, *N* = 35). None of the 20 interaction tests approached significance.

|  | **Congruency × Concreteness** | | **Congruency × Attractiveness** | |
| --- | --- | --- | --- | --- |
| Time Window | Sensor | Source | Sensor | Source |
| 80–130 ms | .471 | .516 | .221 | .465 |
| 150–200 ms | .245 | .755 | .575 | .687 |
| 200–300 ms | .200 | .636 | .184 | .428 |
| 300–600 ms | .527 | .644 | .341 | .617 |
| 600–1000 ms | .322 | .697 | .817 | .671 |


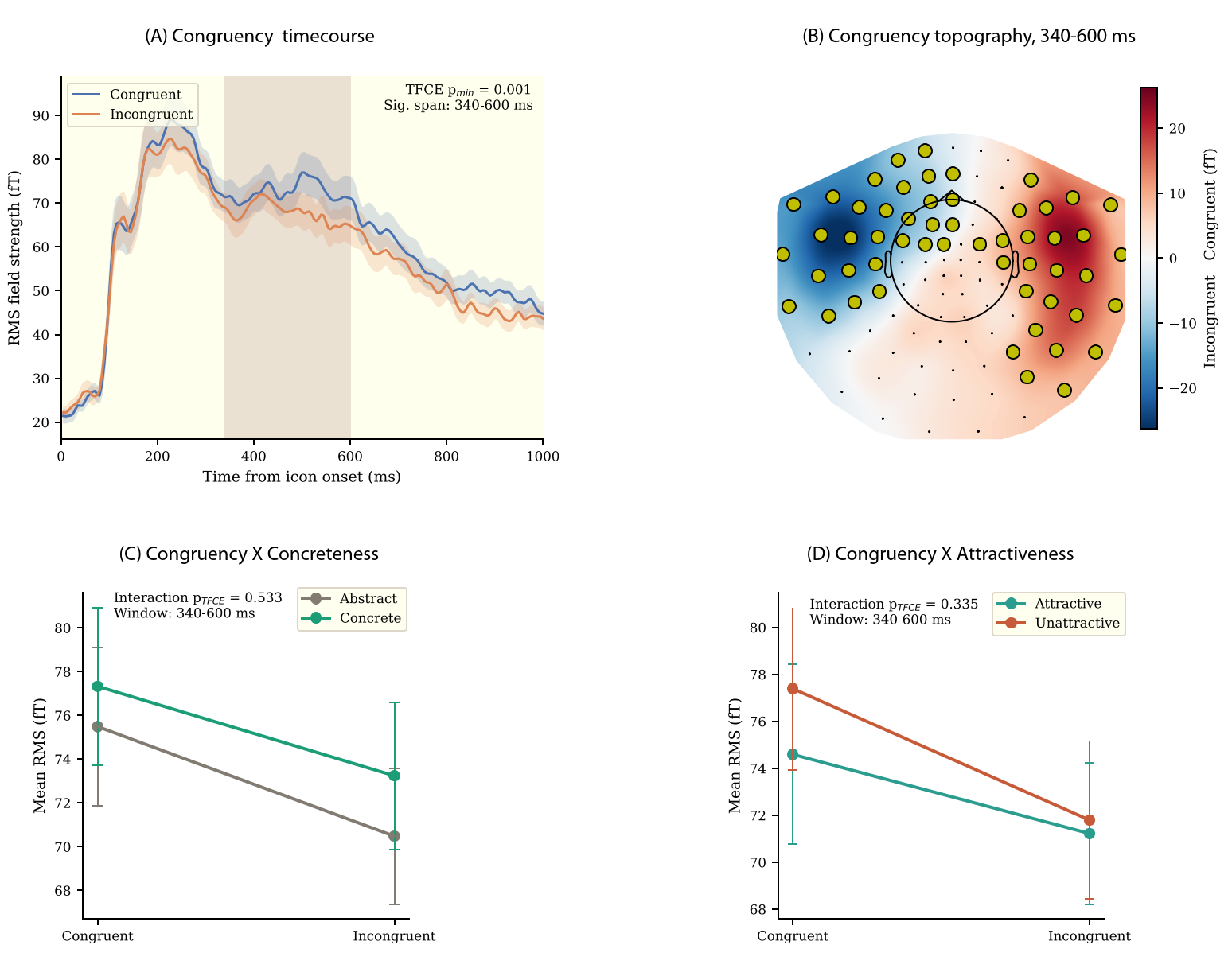


**Figure S6.** Main effect of congruency in the priming-control ANOVAs. (A) Sensor-level RMS timecourse averaged across TFCE-significant magnetometers, with the significant 340-600 ms interval shaded. (B) Topographic map of the incongruent minus congruent difference in that interval; yellow circles mark the TFCE-significant magnetometers. (C, D) Interaction plots from the two 2 x 2 priming-control ANOVAs. In both cases, congruency produced an additive shift without a significant interaction with concreteness (p_TFCE = 0.533) or attractiveness (p_TFCE = 0.335). Error bars represent SEM across subjects. The sensor-level congruency main effect was significant (*p* = 0.001); at source level, the congruency main effect in the 300-600 ms window showed a trend but did not survive correction (Congruency x Concreteness ANOVA: *p* = 0.068; Congruency x Attractiveness ANOVA: *p* = 0.072; N = 35).

**Table S7.** Cluster-averaged descriptive statistics for significant ANOVA effects. Values are given as M ± SEM. Main-effect rows (Fig.S7-1; S7-2) present marginal mean absolute values, collapsed across the other factor and averaged over the exact significant TFCE mask; because absolute values are reported, the direction of the effect is not visible from these numbers alone — spatial patterns are shown in Figure 3. Interaction rows (Fig.S7-3) present the four signed condition cell means averaged over the same significant mask. Sensor-level values are expressed in fT and source-level values in dSPM.

**Table S7-1.** Concreteness Main Effect

| Level | *Sig*. interval | *p*_TFCE,min_ | Partial η² | Abstract | Concrete |
| --- | --- | --- | --- | --- | --- |
| Sensor | 150-190 ms | < .001 | .36 | 83.5 ± 6.3 | 94.9 ± 7.8 |
| Sensor | 210-300 ms | < .001 | .34 | 78.4 ± 6.0 | 71.8 ± 5.6 |
| Sensor | 375-390 ms | .008 | .35 | 42.7 ± 5.1 | 48.3 ± 6.1 |
| Sensor | 610-835 ms | < .001 | .35 | 60.6 ± 7.0 | 58.6 ± 6.5 |
| Source | 90-130 ms | < .001 | .37 | 2.161 ± 0.130 | 2.534 ± 0.170 |
| Source | 150-200 ms | < .001 | .34 | 2.063 ± 0.146 | 1.923 ± 0.121 |
| Source | 200-300 ms | < .001 | .32 | 1.854 ± 0.163 | 1.895 ± 0.144 |
| Source | 300-600 ms | < .001 | .30 | 1.382 ± 0.102 | 1.466 ± 0.089 |
| Source | 600-1000 ms | < .001 | .31 | 1.301 ± 0.090 | 1.193 ± 0.078 |

**Table S7-2.** Attractiveness Main Effect

| Level | *Sig*. interval | *p*_TFCE,min_ | Partial η² | Attractive | Unattractive |
| --- | --- | --- | --- | --- | --- |
| Sensor | 95-130 ms | < .001 | .34 | 77.4 ± 7.5 | 76.5 ± 7.4 |
| Source | 80-130 ms | < .001 | .29 | 2.191 ± 0.144 | 2.092 ± 0.154 |

**Table S7-3.** Concreteness x Attractiveness Interaction

| Level | *Sig*. interval | *p*_TFCE,min_ | Partial η² | Abstract-  Attractive | Abstract-  Unattractive | Concrete-  Attractive | Concrete-  Unattractive |
| --- | --- | --- | --- | --- | --- | --- | --- |
| Sensor | 180-195 ms | .005 | .50 | 49.6 ± 12.8 | 67.3 ± 10.2 | 78.0 ± 11.4 | 51.4 ± 11.5 |
| Source | 100-130 ms | .002 | .76 | -0.119 ± 0.125 | -0.109 ± 0.134 | -0.169 ± 0.150 | 0.109 ± 0.150 |
| Source | 175-185 ms | .006 | .78 | 0.384 ± 0.223 | -0.100 ± 0.207 | -0.326 ± 0.224 | 0.053 ± 0.211 |

**Table S8**. Descriptive statistics for significant source-level RSA effects across time windows. Cells report cluster-averaged mean +/- SD Spearman r across subjects and Cohen’s d; non-significant tests are marked n.s.

## **Table S8-1** Spearman RSA (zero-order)

| Model | 80-130 ms | 150-200 ms | 200-300 ms | 300-600 ms | 600-1000 ms |
| --- | --- | --- | --- | --- | --- |
| Concreteness (0/1) | r=0.007+/-0.005; d=1.29 | r=0.009+/-0.004; d=2.00 | r=0.006+/-0.003; d=1.74 | r=0.004+/-0.002; d=2.68 | r=0.003+/-0.002; d=2.03 |
| Concreteness (1-7) | r=0.009+/-0.008; d=1.11 | r=0.010+/-0.007; d=1.57 | r=0.006+/-0.003; d=1.88 | r=0.005+/-0.002; d=2.11 | r=0.004+/-0.002; d=1.82 |
| Attractiveness (0/1) | r=0.005+/-0.003; d=1.49 | r=0.003+/-0.003; d=1.25 | n.s. | n.s. | r=-0.003+/-0.002; d=-1.38 |
| Attractiveness (1-7)) | n.s. | n.s. | n.s. | n.s. | n.s. |
| Familiarity (0/1) | n.s. | n.s. | n.s. | r=0.015+/-0.021; d=0.74 | n.s. |
| Familiarity (1-7) | n.s. | n.s. | r=0.014+/-0.016; d=0.88 | n.s. | r=0.007+/-0.006; d=1.18 |
| Icon_categoray | r=0.005+/-0.003; d=1.41 | r=0.006+/-0.003; d=2.33 | r=0.005+/-0.002; d=2.29 | r=0.003+/-0.001; d=2.99 | r=0.003+/-0.002; d=1.88 |
| Congruency | n.s. | n.s. | n.s. | r=0.003+/-0.003; d=1.09 | r=0.003+/-0.003; d=1.23 |
| Gabor | r=0.009+/-0.006; d=1.38 | r=0.007+/-0.003; d=2.23 | r=0.005+/-0.003; d=1.63 | r=0.005+/-0.003; d=1.70 | r=0.004+/-0.004; d=1.11 |
| HOG | r=0.006+/-0.003; d=1.83 | r=0.006+/-0.003; d=2.29 | r=0.004+/-0.002; d=2.42 | r=0.003+/-0.001; d=2.56 | r=0.003+/-0.001; d=2.04 |
| Pixel | r=0.007+/-0.005; d=1.39 | r=0.008+/-0.005; d=1.67 | r=0.006+/-0.003; d=1.62 | r=0.004+/-0.002; d=2.09 | r=0.003+/-0.002; d=1.92 |

##

## **Table S8-2** Partial-Spearman RSA (unique contributions)

| Model | 80-130 ms | 150-200 ms | 200-300 ms | 300-600 ms | 600-1000 ms |
| --- | --- | --- | --- | --- | --- |
| Concreteness (1-7) | r=0.009+/-0.006; d=1.35 | r=0.010+/-0.007; d=1.33 | r=0.006+/-0.004; d=1.69 | r=0.005+/-0.002; d=1.99 | r=0.004+/-0.003; d=1.56 |
| Attractiveness (0/1) | n.s. | n.s. | n.s. | n.s. | r=-0.002+/-0.001; d=-1.78 |
| Attractiveness (1-7)) | n.s. | n.s. | n.s. | n.s. | n.s. |
| Familiarity (0/1) | n.s. | n.s. | n.s. | n.s. | n.s. |
| Familiarity (1-7) | n.s. | n.s. | n.s. | n.s. | n.s. |
| Icon_categoray | r=0.004+/-0.003; d=1.11 | r=0.005+/-0.003; d=1.97 | r=0.004+/-0.002; d=2.02 | r=0.003+/-0.001; d=2.68 | n.s. |
| Congruency | n.s. | n.s. | n.s. | r=0.003+/-0.003; d=1.11 | r=0.003+/-0.003; d=1.24 |
| Gabor | r=0.012+/-0.012; d=0.94 | r=0.006+/-0.004; d=1.49 | r=0.008+/-0.009; d=0.96 | n.s. | n.s. |
| HOG | r=0.005+/-0.004; d=1.29 | r=0.005+/-0.004; d=1.13 | r=0.004+/-0.002; d=1.67 | r=0.003+/-0.001; d=2.05 | r=0.003+/-0.002; d=1.31 |
| Pixel | r=0.005+/-0.004; d=1.19 | r=0.006+/-0.005; d=1.24 | r=0.005+/-0.004; d=1.12 | r=0.004+/-0.002; d=1.86 | r=0.004+/-0.002; d=1.68 |


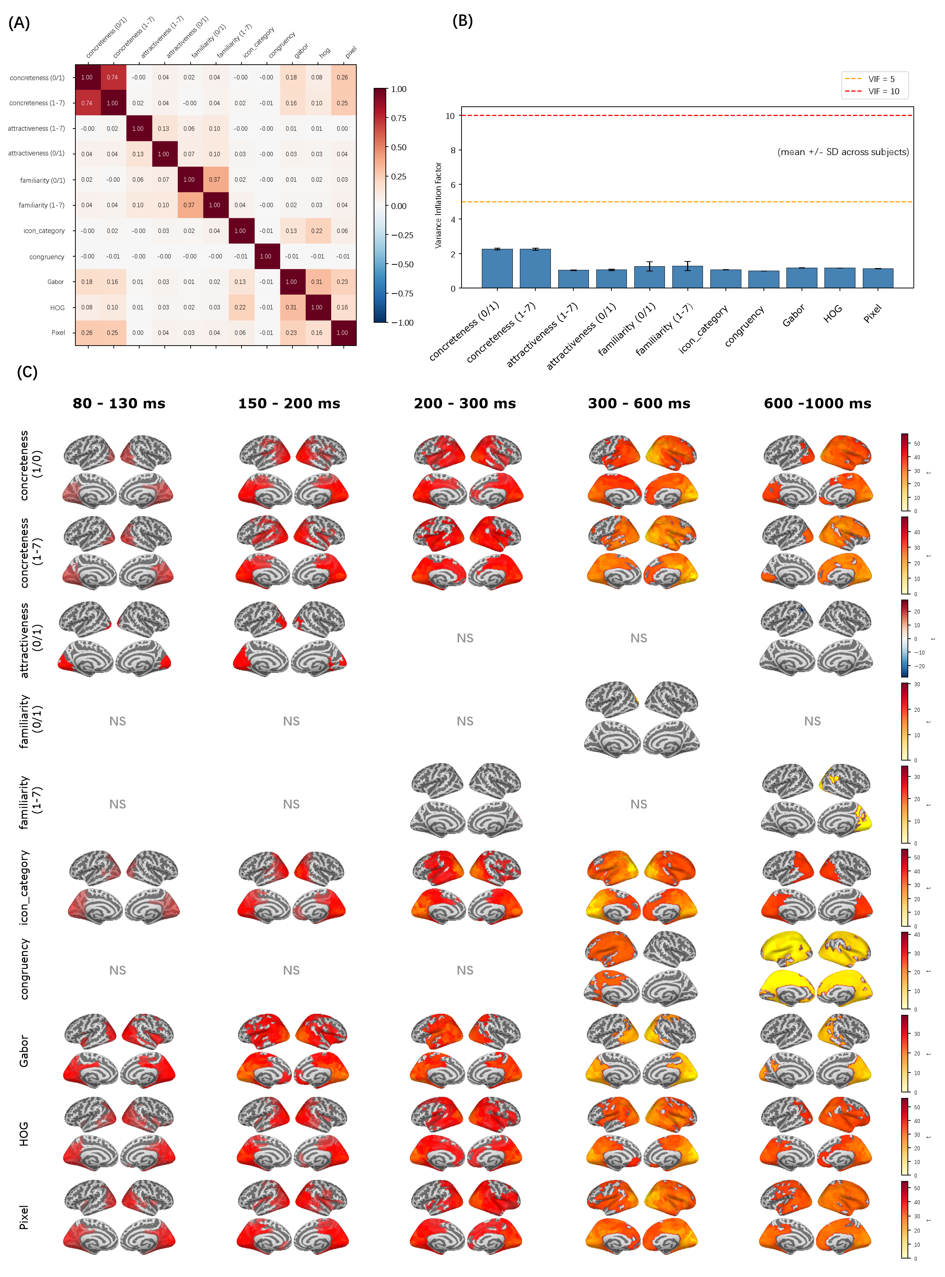


**Figure S7.** RSA model diagnostics and supplementary zero-order RSA source maps. (A) Group-average Spearman correlation matrix for the 11 model representational dissimilarity matrices (RDMs) included in the RSA. Pairwise dependencies were generally low, with the strongest correlation observed between the binary and continuous concreteness RDMs. (B) Variance inflation factors (VIFs) for the same model RDMs (mean ± SD across subjects; *N* = 35). All VIFs were below the conventional cutoff of 5, indicating that multicollinearity was modest and the predictor set was well conditioned. (C) Source-level brain maps from the supplementary zero-order Spearman RSA across the five predefined time windows (80–130, 150–200, 200–300, 300–600, and 600–1000 ms). Rows correspond to model RDMs showing at least one significant effect, and columns correspond to time windows; cells labeled n.s. were not significant. Maps show ROIs surviving the Bonferroni-corrected TFCE threshold (α = .01), rendered in lateral and medial views of both hemispheres. Warm colors indicate positive RSA effects and cool colors indicate negative effects; color bars denote t-TFCE values.

**Table S9.** Summary of predefined time windows and their functional interpretations

| **Time Window** | **Time Range (ms)** | **GFP Peak (ms)** | **Associated ERP/ERF Components** | **Functional Interpretation** | **Key references** |
| --- | --- | --- | --- | --- | --- |
| TW1 | 80-130 | ~105 | P1/M100 | Early feedforward visual encoding sensitive to low-level visual features | Di Russo et al., 2002; Herrmann et al., 2005; Liu et al., 2002; Luck, 2014 |
| TW2 | 150-200 | ~175 | N170 / M170 | Configural and category-level processing, including structural encoding | Bentin et al., 1996; Halgren et al., 2000; Rossion & Jacques, 2008 |
| TW3 | 200-300 | ~250 | N250 / N300 | Transition from perceptual processing to semantic activation | Schendan & Kutas, 2002, 2003; Tanaka et al., 2006 |
| TW4 | 300-600 | ~450 | N400 / LPC | Semantic integration and memory retrieval processes | Kutas & Federmeier, 2011; Rugg & Curran, 2007 |
| TW5 | 600-1000 | ~800 | LPC | Late sustained processing related to evaluative and decision-related processes | Friedman, 1990; Woodruff et al., 2006; Voss & Paller, 2009 |
